# Emerging rodent-associated *Bartonella*: a threat for human health?

**DOI:** 10.1186/s13071-022-05162-5

**Published:** 2022-03-31

**Authors:** Maria Krügel, Nina Król, Volkhard A. J. Kempf, Martin Pfeffer, Anna Obiegala

**Affiliations:** 1 Institute of Animal Hygiene and Veterinary Public Health, University of Leipzig, An den Tierkliniken 1, 04103 Leipzig, Germany; 2 Institute for Medical Microbiology and Infection Control, University Hospital, Goethe University, Frankfurt am Main, Germany; 3 National Consiliary Laboratory for Bartonella, Frankfurt am Main, Germany

**Keywords:** Rodents, Host association, *Candidatus* species, *Bartonella*, Small mammal, Lagomorphs, Taxon

## Abstract

**Background:**

Species of the genus *Bartonella* are facultative intracellular alphaproteobacteria with zoonotic potential. *Bartonella* infections in humans range from mild with unspecific symptoms to life threatening, and can be transmitted via arthropod vectors or through direct contact with infected hosts, although the latter mode of transmission is rare. Among the small mammals that harbour *Bartonella* spp., rodents are the most speciose group and harbour the highest diversity of these parasites. Human–rodent interactions are not unlikely as many rodent species live in proximity to humans. However, a surprisingly low number of clinical cases of bartonellosis related to rodent-associated *Bartonella* spp. have thus far been recorded in humans.

**Methods:**

The main purpose of this review is to determine explanatory factors for this unexpected finding, by taking a closer look at published clinical cases of bartonellosis connected with rodent-associated *Bartonella* species, some of which have been newly described in recent years. Thus, another focus of this review are these recently proposed species.

**Conclusions:**

Worldwide, only 24 cases of bartonellosis caused by rodent-associated bartonellae have been reported in humans. Possible reasons for this low number of cases in comparison to the high prevalences of *Bartonella* in small mammal species are (i) a lack of awareness amongst physicians of *Bartonella* infections in humans in general, and especially those caused by rodent-associated bartonellae; and (ii) a frequent lack of the sophisticated equipment required for the confirmation of *Bartonella* infections in laboratories that undertake routine diagnostic testing. As regards recently described *Bartonella* spp., there are presently 14 rodent-associated *Candidatus* taxa. In contrast to species which have been taxonomically classified, there is no official process for the review of proposed *Candidatus* species and their names before they are published. This had led to the use of malformed names that are not based on the International Code of Nomenclature of Prokaryotes. Researchers are thus encouraged to propose *Candidatus* names to the International Committee on Systematics of Prokaryotes for approval before publishing them, and only to propose new species of *Bartonella* when the relevant datasets allow them to be clearly differentiated from known species and subspecies.

**Graphical Abstract:**

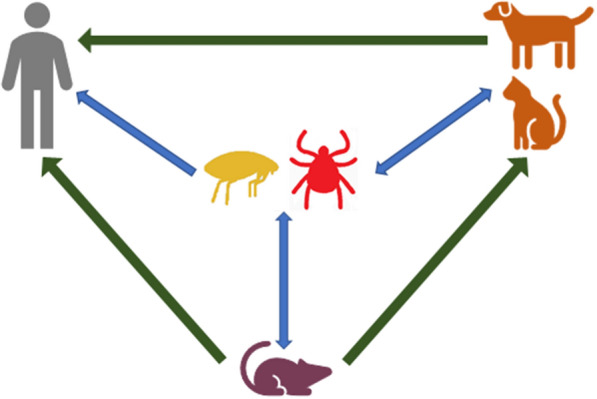

**Supplementary Information:**

The online version contains supplementary material available at 10.1186/s13071-022-05162-5.

## Background

The genus *Bartonella* (family Bartonellaceae; order Rhizobiales) comprises facultative intracellular alphaproteobacteria. An increasing number of *Bartonella* species are recognized as zoonotic pathogens [1]. In humans, bartonellosis can have a variety of mild and unspecific clinical signs and symptoms, but can also be life threatening [2, 3]. Bartonellae can be transmitted to humans indirectly from blood-sucking arthropod vectors through the scratches of an infected reservoir host (e.g. cats) or via contact with infectious faeces of arthropod vectors. Fleas play a major role in the transmission of *Bartonella*, especially the cat flea (*Ctenocephalides felis*), one of the most common flea species in central Europe, which is host opportunistic, and thus a common source of infection of *Bartonella*, and especially *Bartonella henselae* [4]. Direct transmission through contact with reservoir hosts can not be excluded as a possible transmission path, although it is considered highly unlikely.

A wide variety of mammals are suspected of being reservoir hosts of *Bartonella* spp. [2]. Among mammals, small mammals, including bat and rodent species, are the group that harbours by far the highest diversity of *Bartonella* spp. [5]. Moreover, high prevalences of *Bartonella* spp. have been detected in rodents, the most speciose group of mammals [6]. As many rodent species live in proximity to humans in many parts of the world, human-rodent interactions are not unlikely. Nonetheless, very low numbers of clinical cases of bartonellosis in humans have been reported in the context of rodent-associated *Bartonella*. Thus, the purpose of this review is to determine explanatory factors for this unexpected finding. Therefore, we decided to explore (i) potential risk factors for humans; (ii) clinical cases described in recent years connected with rodent-associated bartonellae; and, additionally (iii) the growing trend in the scientific literature of newly described *Bartonella* taxa, including the reporting of a high number of *Candidatus* species. This review is focussed on rodent, lagomorph and other small mammal species (with the exception of bats) as they may be sympatric and share the same ectoparasites and *Bartonella* species.

## Current knowledge on rodent reservoirs of *Bartonella* spp.

### Global distribution of* Bartonella* spp. in small mammals

**Fig. 1:**
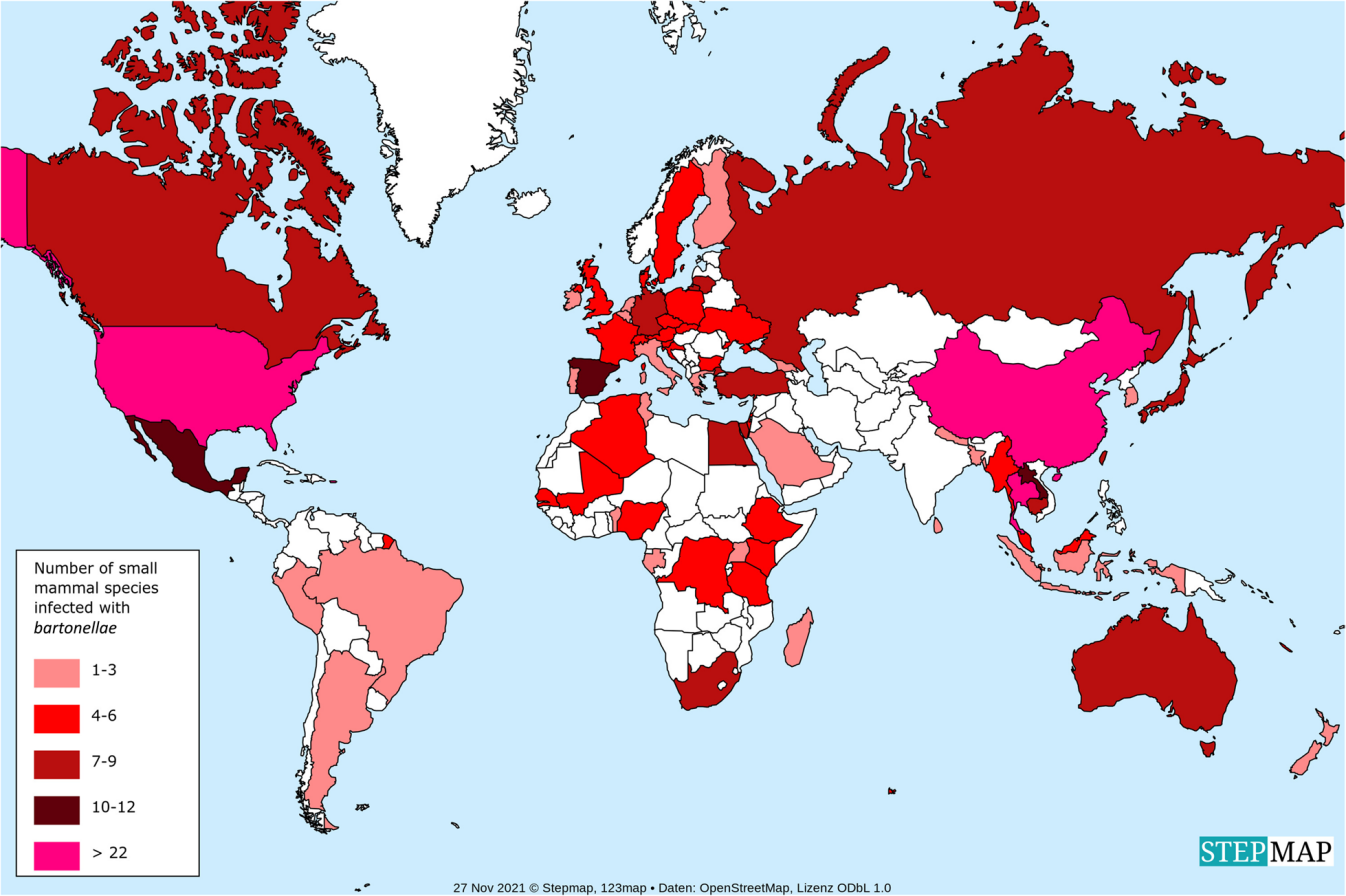
The number of *Bartonella*-positive small mammal species per country (listed in Additional file 1: Table S1)

Rodent-associated bartonellae are distributed worldwide and have been the subject of research on almost every continent. For a systematic review of papers describing the detection of *Bartonella* in small mammal species, the following search engines were used: Google Scholar, PubMed, and Google. The following terms were searched for, solely or in combination: ‘*Bartonella*’; ‘rodent’; ‘small mammal’; and, in addition [name of any country in the world]; or [name of any continent in the world]. Furthermore, a separate search was conducted with the search term [name of any small mammal genus] in combination with one of the following terms: [*Bartonella*]; [Bartonellae]; [Bartonellosis]. Only studies published in the English language and in peer-reviewed journals were taken into consideration. In total, 132 studies were included in the analysis, representing research on *Bartonella* in a total of 231 small mammal species and subspecies (excluding bats). Research on *Bartonella* in small mammals has been conducted in 67 of the 195 countries (34.4%) of the world. Most of these studies were conducted in North America [in both Canada and the USA (100%)], followed by Europe [25 out of 44 countries (54.6%)] and Asia [19 out of 48 countries (39.6%)]. The continents/regions for which the lowest numbers of studies were reported are as follows: Africa [15 out of 54 countries (27.8%)], Oceania [two out of 14 countries (14.3%)], and Latin America and the Carribean [four out of 33 countries (12.1%)] (Additional file 1: Table S1). Thus, small mammals in a large number of countries have not yet been investigated for the presence of *Bartonella* spp. Most of these countries are located in Latin America, the Middle East, and Central Africa, and the lack of published data from them might be partly due to their economic and/or political situation. However, it is considered important that studies are especially carried out in countries in Central Africa, as they are among those with the lowest health coverage [7]. People from these areas make up a large proportion of those who most frequently need treatment for neglected tropical diseases [7], and infections with rodent-borne *Bartonella* spp. can also be expected to occur more frequently in these areas. It is also worth noting that studies were more frequently conducted in some countries than in others (e.g. there were 17 from the USA but only one from Argentina). The studies undertaken in the USA were conducted thoroughly and, in total, reported 25 small mammal species positive for* Bartonella* (Fig. 1).

The most frequently studied genus was *Rattus*, and in particular the two cosmopolitan species *Rattus norvegicus* and *Rattus rattus*. Members of the genera *Apodemus*, *Bandicota*, *Microtus*, *Mus*, and *Myodes* were very often associated with *Bartonella* spp*.* The most studied rodent species was *R. norvegicus* (43 studies from four of the seven continents), followed by *R. rattus* (41 studies), *Mus musculus* (25 studies) and *Clethrionomys glareolus* (24 studies). The five *Bartonella* species most frequently detected in small mammal hosts were *Bartonella grahamii* (found in 53 small mammal species, and in 31 countries), *Bartonella elizabethae* (found in 43 small mammal species, and in 34 countries), *Bartonella tribocorum* (found in 30 small mammal species, and in 27 countries), *Bartonella taylorii* (found in 27 small mammal species, and in 21 countries), and *Bartonella queenslandensis* (found in 29 small mammal species, and in 13 countries). Small mammals positive for *Bartonella* were found in 65 (97.0%) of 67 investigated countries (they were not found in Hungary and Pakistan). The five most frequently listed *Bartonella* species in Additional file 1: Table S1 may, however, be a distortion as, for example, *B. elizabethae* is one of the first rodent-associated species to have been described (in 1993) whereas other rodent-associated species such as *B. kosoyi* were not described until much later (in 2018). Furthermore, it should be noted that various methods were used in the studies, and that not all the published sequences had a homology of 100%.

## Clinical cases of bartonellosis in humans in the context of rodent-associated *Bartonella* spp.

### Clinical symptoms/clinical cases, diagnostics, and pathogenicity

The most frequently described *Bartonella* species pathogenic for humans include the human-specific species *Bartonella bacilliformis* (transmitted via sand flies), which causes Carrion’s disease in South America; the zoonotic, cat-transmitted species *B. henselae*, which is responsible for cat scratch disease; and the human-specific species *Bartonella quintana* (transmitted via body lice), the causative agent of trench fever [8]. Much less is known about human infections with other *Bartonella* spp. In general, endocarditis, lymphadenopathy and neuroretinitis are common symptoms of severe cases of bartonellosis [9]. A detailed PubMed analysis (performed on 2 October 2021) with the search terms ‘*Bartonella* [species]’ where the species was one of the 33 *Bartonella* species given in Table 1 [e.g. (‘*B. alsatica*’) and (‘infection’)] revealed only 14 publications citing evidence for human infections (see Table 2).

**Table 1:** Published *Bartonella* species and subspecies, the status of their published name according to the International Code of Nomenclature of Prokaryotes (*ICNP*), year first described, and first-mentioned host reservoir(s) together with their category and taxonomic order

Order	Host species	Host category	Species name	Nomenclatural status	Candidatus status	Reviewed name	Former name	References	Year
Rodentia	*Acomys russatus*	Rodent	*Bartonella acomydis*	Validly published under the ICNP	No	Yes		[51]	2013
	*Apodemus* spp*.*	Rodent	*Bartonella birtlesii*	Validly published under the ICNP	No	Yes		[175]	2000
	*Callosciurus notatus*	Rodent	*Bartonella callosciuri*	Validly published under the ICNP	No	Yes		[51]	2013
	*Rattus leucopus*	Rodent	*Bartonella coopersplainsensis*	Validly published under the ICNP	No	Yes		[136]	2009
	*Microtus agrestis*	Rodent	*Bartonella doshiae*	Validly published under the ICNP	No	Yes		[176]	1995
	Rodents	Rodent	*Bartonella elizabethae*	Validly published under the ICNP	No	Yes	*Rochalimaea elizabethae*	[177]	1993
	*Dipodillus dasyurus*	Rodent	*Bartonella fadhilii*	Not validly published	Yes	Yes (corrected)	*Bartonella fadhilae*	[178]	2017
	*Microtus* spp*.*	Rodent	*Bartonella fuyuanensis*	Validly published under the ICNP	No	Yes		[62]	2015
	*Lophuromys* sp*.*	Rodent	*Bartonella gabonensis*	Not validly published	No	No		[179]	2020
	*Gerbillus* spp.	Rodent	*Bartonella gerbillinarum*	Not validly published	Yes	Yes		[180]	2018
	*Clethrionomys glareolus*	Rodent	*Bartonella grahamii*	Validly published under the ICNP	No	Yes		[176]	1995
	Rodents	Rodent	*Bartonella heixiaziensis*	Validly published under the ICNP	No	Yes		[62]	2015
	*Jaculus orientalis*	Rodent	*Bartonella jaculi*	Validly published under the ICNP	No	Yes		[51]	2013
	*Apodemus argenteus*	Rodent	*Bartonella japonica*	Validly published under the ICNP	No	Yes		[80]	2010
	*Gerbillus* spp.	Rodent	*Bartonella khokhloviae*	Not validly published	Yes	Yes (corrected)	*Bartonella khokhlovae*	[180]	2018
	*Rattus rattus*	Rodent	*Bartonella kosoyi*	Validly published under the ICNP	No	Yes		[181]	2020
	*Rattus rattus*	Rodent	*Bartonella krasnovii*	Validly published under the ICNP	No	Yes		[181]	2020
	*Marmota monax*	Rodent	*Bartonella marmotae*	Not validly published	Yes	Yes (corrected)	*Bartonella monaxi*	[182]	2009
	*Mastomys erythroleucus*	Rodent	*Bartonella mastomysi*	Not validly published	No	No	*Bartonella mastomydis*	[183]	2018
	*Gerbillus* spp.	Rodent	*Bartonella negevensis*	Not validly published	Yes	Yes (corrected)	*Bartonella negeviensis*	[180]	2018
	*Pachyuromys duprasi*	Rodent	*Bartonella pachyuromydis*	Validly published under the ICNP	No	Yes		[51]	2013
	*Peromyscus* spp.	Rodent	*Bartonella peromysci*	Validly published under the ICNP	No	Yes		[176]	1995
	*Rattus norvegicus*	Rodent	*Bartonella phoceensis*	Not validly published	No	No		[160]	2004
	*Melomys spp.; Rattus* spp.	Rodent	*Bartonella queenslandensis*	Validly published under the ICNP	No	Yes		[136]	2009
	*Mastomys erythroleucus*	Rodent	*Bartonella raoultii*	Not validly published	Yes	No		[131]	2014
	*Rattus* spp.	Rodent	*Bartonella rattaustraliani*	Validly published under the ICNP	No	Yes		[136]	2009
	*Rattus norvegicus*	Rodent	*Bartonella rattimassiliensis*	Not validly published	No	No		[160]	2004
	*Sciurus vulgaris*	Rodent	*Bartonella rudakovii*	Not validly published	Yes	Yes		[184]	2012
	*Gerbilliscus gambianus*	Rodent	*Bartonella sahelensis*	Not validly published	No	Yes (corrected)	*Bartonella saheliensis*	[131]	2014
	*Dipodillus dasyurus*	Rodent	*Bartonella sanaae*	Not validly published	Yes	Yes		[178]	2017
	*Apodemus argenteus*	Rodent	*Bartonella silvatica*	Validly published under the ICNP	No	Yes		[80]	2010
	*Apodemus, Clethrionomys*	Rodent	*Bartonella taylorii*	Validly published under the ICNP	No	Yes		[176]	1995
	*Rattus surifer*	Rodent	*Bartonella thailandensis*	Not validly published	Yes	Yes		[114]	2009
	*Rattus* spp.	Rodent	*Bartonella tribocorum*	Validly published under the ICNP	No	Yes		[185]	1998
	Voles	Rodent	*Bartonella vinsonii* subsp.* vinsonii*	Validly published under the ICNP	No	Yes		[186]	1996
	*Peromyscus yucatanicus*	Rodent	*Bartonella vinsonii* subsp.* yucatanensis*	Not validly published	No	No		[187]	2016
	*Glaucomys volans*	Rodent	*Bartonella volans*	Not validly published	Yes	Yes		[182]	2009
	*Cynomys ludovicianus*	Rodent	*Bartonella washoensis* subsp.* cynomyisi*	Not validly published	Yes	Yes (corrected)	*Bartonella washoensis* subsp.* cynomyisii*	[188]	2008
Lagomorpha	*Oryctolagus cuniculus*	Small mammal (rabbit)	*Bartonella alsatica*	Validly published under the ICNP	No	Yes		[185]	1999
Dasyuromorphia	*Antechinus flavipes*	Small mammal (Marsupialia)	*Bartonella antechini*	Not validly published	Yes	Yes		[189]	2011
Diprotodontia	*Macropus giganteus*	Big mammal (Marsupialia)	*Bartonella australis*	Not validly published	No	No		[190]	2007
	*Bettongia penicillata*	Small mammal (Marsupialia)	*Bartonella bettongiae*	Not validly published	Yes	Yes (corrected)	*Bartonella woyliei*	[189]	2011
Eulipotyphla	*Crocidura russula*	Small mammal (Soricidae)	*Bartonella florencae*	Validly published under the ICNP	No	Yes (corrected)	*Bartonella florenciae*	[131]	2014
	*Crocidura suaveolens*	Small mammal (Soricidae)	*Bartonella refiksaydamii*	Not validly published	No	No		[191]	2021
	*Talpa europaea*	Small mammal (Talpidae)	*Bartonella talpae*	Validly published under the ICNP	No	Yes		[176]	1995
Chiroptera	*Myotis daubentonii*	Small mammal (Vespertilionidae)	*Bartonella hemsundetensis*	Not validly published	Yes	Yes (corrected)	*Bartonella hemsundetiensis*	[192]	2015
	Bats	Small mammal	*Bartonella lascolai*	Not validly published	Yes	No		[193]	2016
	Bats	Small mammal	*Bartonella naantaliensis*	Not validly published	Yes	No		[194]	2014
	Bats	Small mammal	*Bartonella rolaini*	Not validly published	Yes	No		[193]	2016
	Fruit bats	Small mammal	*Bartonella rousetti*	Not validly published	No	Yes		[188, 195]	2020
Peramelemorphia	*Perameles bougainville*	Small mammal (Peramelemorphia)	*Bartonella peramelis*	Not validly published	yes	Yes (corrected)	*Bartonella bandicootii*	[189]	2011
Xenarthra	NA	Small mammal	*Bartonella washoensis* subsp.* brasiliensis*	Not validly published	Yes	Yes		[196]	2020
Primates	*Homo sapiens sapiens*	Human	*Bartonella bacilliformis*	Validly published under the ICNP	No	Yes		[197]	1913
	*Homo sapiens sapiens*	Human	*Bartonella mayotimonensis*	Not validly published	Yes	Yes		[143]	2010
	*Homo sapiens sapiens*	Human	*Bartonella quintana*	Validly published under the ICNP	No	Yes		[177]	1993
	*Homo sapiens sapiens*	Human	*Bartonella tamiae*	Not validly published	No	No		[198]	2008
	*Homo sapiens sapiens*	Human	*Bartonella vinsonii* subsp.* arupensis*	Validly published under the ICNP	No	Yes		[199]	1999
	*Canis familiaris/Homo sapiens sapiens*	Human	*Bartonella vinsonii*	Validly published under the ICNP	No	Yes		[177]	1993
	*Homo sapiens sapiens*	Human	*Bartonella washoensis*	Not validly published	No	Yes (corrected)	*Bartonella washoeensis*	[200]	1998
Carnivora	*Felis silvestris catus*	Big mammal	*Bartonella clarridgeiae*	Validly published under the ICNP	No	Yes		[201]	1996
	*Felis silvestris catus*	Big mammal	*Bartonella henselae*	Validly published under the ICNP	No	Yes		[177]	1993
	*felis silvestris catus*	Big mammal	*Bartonella koehlerae*	Validly published under the ICNP	No	Yes		[202]	2000
	*Puma concolor, Lynx rufus*	Big mammal	*Bartonella koehlerae* subsp.* bothieri*	Not validly published	No	No		[203]	2016
	*Puma concolor, Lynx rufus*	Big mammal	*Bartonella koehlerae* subsp.* boulouisii*	Not validly published	No	No		[203]	2016
	*Felis silvestris catus*	Big mammal	*Bartonella koehlerae* subsp.* koehlerae*	Not validly published	No	No		[202]	2000
	*Procyon lotor, Canis familiaris*	Big mammal	*Bartonella rochalimae*	Validly published under the ICNP	No	Yes		[204]	2012
	*Canis familiaris*	Big mammal	*Bartonella merieuxii*	Not validly published	Yes	Yes		[184]	2012
	*Canis familiaris*	Big mammal	*Bartonella vinsonii* subsp.* berkhoffii*	Validly published under the ICNP	No	Yes		[186]	1996
	*Urva auropunctata*	Big mammal	*Bartonella kittensis*	Not validly published	Yes	No		[205]	2021
Artiodactyla	*Bos taurus*	Big mammal	*Bartonella bovis*	Validly published under the ICNP	No	Yes	*Bartonella weissii*	[206]	2002
	*Capreolus capreolus*	Big mammal	*Bartonella capreoli*	Validly published under the ICNP	No	Yes		[206]	2003
	*Bos taurus*	Big mammal	*Bartonella chomelii*	Validly published under the ICNP	No	Yes		[207]	2004
	*Bos taurus*	Big mammal	*Bartonella davoustii*	Not validly published	Yes	Yes (corrected)	*Bartonella davousti*	[208]	2017
	*Camelus dromedarius*	Big mammal	*Bartonella dromedarii*	Not validly published	No	No		[209]	2014
	*Ovis aries*	Big mammal	*Bartonella melophagi*	Not validly published	No	Yes		[210, 211]	2016
	*Ovis aries*	Big mammal	*Bartonella ovis*	Not validly published	Yes	Yes		[212]	2018
	*Odocoileus virginianus*	Big mammal	*Bartonella odocoilei*	Not validly published	Yes	No		[213]	2021
	*Bos taurus, Capreolus capreolus*	Big mammal	*Bartonella schoenbuchensis*	Validly published under the ICNP	No	Yes (corrected)	*Bartonella schoenbuchii*	[214]	2001
	*Eratyrus mucronatus*	Big mammal	*Bartonella cariotis*	Not validly published	Yes	Yes (corrected)	*Bartonella rondoniensis*	[215]	2017
Ixodida	*Ornithodoros sonrai*	Arthropoda	*Bartonella senegalensis*	Validly published under the ICNP	No	Yes		[131]	2014
	*Ornithodoros sonrai*	Arthropoda	*Bartonella massiliensis*	Not validly published	No	No		[216]	2019
Siphonaptera	*Orchopeas howardi*	Arthropoda	*Bartonella durdenii*	Not validly published	Yes	Yes		[182]	2009
Hymenoptera	*Apis mellifera*	Arthropoda	*Bartonella apis*	Validly published under the ICNP	No	Yes		[217]	2016
ND	ND	Arthropoda	*Bartonella ancashensis*	Validly published under the ICNP	No	Yes (corrected)	*Bartonella ancashi*	[218]	2015

*Subsp*. Subspecies, *ND* not determined

**Table 2:** Human infections by small mammal-associated *Bartonella* spp.

*Bartonella* spp.	Clinical disease	Patient details	Microbiological diagnosis	Antimicrobial therapy	Clinical outcome	References
*Bartonella alsatica*	Prosthetic vascular graft infection	Male, 66 years old, hunter	PCRs (biopsies), sequence analysis	Doxycycline 2 × 100 mg/day (6 months)	Improvement of renal function (no comprehensible link to antibiotic treatment)	[219]
	Endocarditis	Female, 77 years old, rabbit breeder	Serology (not standardized)^a^, culture and PCR (blood) negative	Gentamicin (15 days), amoxicillin (6 weeks)	Clinical improvement, no details given	[220]
	Lymphadenopathy	Female, 79 years old, rabbit butcher	PCRs (lymph node), sequence analysis, serology (not standardized)^a^, histology (unspecific)^b^	Doxycycline 200 mg/day (3 weeks)	Surgical removal of lymph nodes, no further details given	[115]
	Endocarditis	Male, 74 years old, bioprosthetic aortic valve, parotideal cancer	Shell vial culture, PCR (valves and blood), sequence analysis, histology (unspecific)^b^	Amoxicillin 12 g/day, gentamicin 320 mg/day changed to doxycycline 200 mg/day (6 weeks), ceftriaxone 2 g/day	Valve replacement, patient became apyretic	[221]
*Bartonella doshiae*	Unspecific (fatigue, blurred vision, arthralgia)	Female, 45 years old	Prolonged cultivation, PCR detection from blood	NA	NA	[222]
*Bartonella elizabethae*	Bacillary angiomatosis	Male, 35 years old, human immunodeficiency virus-positive	PCR (biopsy), sequence analysis, histology (unspecific)^b^	NA	No patient follow-up (patient incompliance)	[223]
	Acute febrile illness	Patients from rural Thailand (*n* = 2/14)	PCR from shell vial cultures, sequence analysis	NA	NA	[224]
	Lymphadenopathy	Female, 18 years old, culture negative	PCRs (lymph node)	Azithromycin 3 × 250 mg/day, duration NA	Restitutio ad integrum	[225]
*Bartonella grahamii*	Lymphadenopathy	Female, 57 years old, cat scratch (exposed to infected rodents), leukaemia and bone marrow transplantation	Several PCRs (lymph node) and sequence analysis, histology (unspecific)^b^	Azithromycin 250 mg/day (5 weeks)	Clinical restitutio ad integrum, no abnormal findings by ultrasound examination	[25]
*B. grahamii*^c^	Neuroretinitis	Male, 55 years old, dog owner	PCRs (anterior chamber fluid), sequence analysis, serology (not standardized)^a^	Doxycycline 200 mg/day, rifampin 600 mg/day	Intraocular inflammation extinguished, cataract development	[226]
*Bartonella rattimassiliensis*	Acute febrile illness	Patients from rural Thailand (*n* = 1)	PCR from shell vial cultures, sequence analysis	NA	NA	[224]
*Bartonella tribocorum*	Acute febrile illness, unspecific (fatigue, muscle pain, headache)	Patients from rural Thailand (*n* = 1)	Shell vial culture, PCR (blood) and sequence analysis	NA	NA	[224]
		Male, 64 years old, dog owner	Prolonged cultivation, PCR detection from blood	NA	NA	[222]
*Bartonella vinsonii* subsp. *vinsonii*	Acute febrile illness	Patients from rural Thailand (*n* = 1)	PCR from shell vial cultures, sequence analysis	NA	NA	[224]
	Blood stream infection (fever, unspecific neuropsychiatric symptoms)	Female, 14 years old	Pre-enriched media PCRs (blood), sequence analysis	Doxycycline (2 months), clarithromycin (2 months), rifampin (2 months), no success	Minimal symptomatic improvement	[227]
*Bartonella vinsonii *subsp. *arupensis*	Acute febrile illness	Patients from rural Thailand (*n* = 2)	PCR from shell vial cultures, sequence analysis	NA	NA	[224]
	Acute febrile illness, unspecific (fever, myalgia, fatigue), elevated liver enzymes	Patients from rural Thailand (*n* = 4)	Pre-enriched media PCR, sequence analysis	NA	NA	[228]
	Hepatic granulomatous lesions	Female, 11 years old, cat exposure	PCR (liver biopsy, cat blood)	Azithromycin 500 mg (4 weeks) followed by doxycycline 2 × 100 mg (four weeks)	Clinical improvement (decrease in size of hepatic lesions)	[229]
	Endocarditis	Male, 79 years old, bioprosthetic aortic valve	Serology (non-standardized)^a^, PCR, (serum) sequence analysis	Doxycycline 200 mg/day, ofloxacin 200 mg/day, duration NA	Clinical improvement (no details given)	[230]

*n* Number of patients examined; *NA* not available

^a^Experimental approach; applied serology not evaluated according to laboratory diagnostic standards

^b^Detection of structures under Warthin–Starry staining or similar staining (no specific staining for *Bartonella* spp.)

^c^Species identification questionable, more likely *Bartonella tribocorum* [25]

When high diagnostic standards were applied (including direct pathogen detection via culture or PCR), only eight *Bartonella* species or subspecies (*Bartonella vinsonii* subsp. *arupensis* infections, *B. elizabethae* infections, *Bartonella alsatica* infections, *B. tribocorum* or *B. vinsonii* subsp. *vinsonii* infections,* Bartonella doshiae*,* B. grahamii*,* Bartonella rattimassiliensis*) were reported (in total, 24 confirmed patient cases; see Table 2).

The analysis of the frequencies of clinical entities showed that 12 patients suffered from acute febrile illness (most likely associated with bacteremia/blood stream infections) (50%), three patients from endocarditis or prosthetic valvular graft infections (12.5%), three patients from lymphadenopathy (12.5%), two from nonspecific symptoms (8.3%), and one each from bacillary angiomatosis, hepatic lesions or neuroretinitis (4.2%). Two of these patients were immunocompromised (human immunodeficiency virus infection, leukemia); no clear association with an underlying comorbidity was reported for the remaining patients. The reported antibiotic therapy regime varied but often included the administration of a macrolide combined with doxycycline for some weeks, which often resulted in clinical improvement.

From a clinical point of view, ‘acute febrile illness’ and ‘endocarditis’ can be classified as ‘bacteremia/blood stream infections’, which 15 of 24 reported patients (62.5%) suffered from. Although these cases were anecdotal, it can be suggested that human infections by rodent-associated *Bartonella* spp. are rare. To our knowledge, there are several possible reasons for this low number of case reports: (i) physicians are very likely unaware of *Bartonella* infections (especially when rodent-associated), and thus do not include them in their differential diagnosis; (ii) laboratories may not able to detect these pathogens due to their fastidious nature, and because their diagnostic portfolio does not include PCR tests for the detection of *Bartonella* spp. or they do not carry out long, sterile microaerophilic incubations for the cultivation of samples from patients. Moreover, (iii) bartonellosis might only cause mild and unspecific symptoms; and (iv) rodent-associated *Bartonella* infections of humans may simply be rare medical entities. A possible molecular explanation for the latter is the host restriction of *Bartonella* species mediated by their respective Trw type IV secretion systems (T4SSs) [10]. The Trw T4SS (originally described as a plasmid conjugation system) is crucial for adhesion to erythrocytes and subsequent erythrocyte invasion, and *Bartonella* with mutations in the* trwE* gene (signature-tagged mutagenesis) are unable to establish long-lasting bacteremia in certain rodent infection models. It has been demonstrated that the Trw systems of certain *Bartonella* spp. are responsible for species-specific host-restricted adhesion to erythrocytes. For instance, the Trw T4SS of *B. tribocorum* mediates a significant bacterial infection in Wistar rats, but infection human erythrocytes is 23 times less efficient. It seems likely that infections of humans by rodent-adapted *Bartonella* spp. rarely occur because the rodent-pathogen Trw system and human erythrocyte host receptors simply do not match.

### Groups at risk of* Bartonella* infection in the context of rodent-associated* Bartonella* spp.

Many *Bartonella* species are pathogenic for humans. However, *B. henselae*, *B. bacilliformis* and *B. quintana* cause most cases of *Bartonella* disease in humans [8, 11]. Veterinarians, veterinary nurses and people that work with and care for animals seem to be at increased risk of infection as they are particularly exposed to reservoir hosts and vectors of *Bartonella* spp. [12–14]. Oteo et al. [15] found that 11.2–56% of tested veterinary professionals in Spain showed seroreactivity for *B. henselae*, *B. quintana*, and/or *B. vinsonii berkhoffii*. *Bartonella* spp. were even isolated from 7.9% of the positive individuals, although all of them were asymptomatic [15]. *Bartonella henselae* is also reported to have possibly contributed to the death of two veterinarians [16]. Cat and dog owners also appear to be at increased risk of infection. Transmission of *B. henselae* is associated with scratches received from both cats and dogs [17]. Owners of a cat ≤ 12 months old have an increased risk of infection with *B. henselae* compared to those with a cat > 12 months old [18]. Forest workers and orienteers seem to be the other groups at risk [19, 20]. Furthermore, a higher risk of infection has also been described for homeless people, alcoholics, and drug addicts who administer substances intravenously [21, 22]. Though an intravenous transmission route seems unlikely, one study did show that drug addicts who administered substances intravenously were more at risk of contracting *Bartonella* spp. [20]. Infestation with ectoparasites such as lice and fleas due to poor hygiene may also lead to bartonellosis, especially in homeless people [23].

The risk factors for rodent-associated *Bartonella* infections in humans are similar to those mentioned above for *Bartonella* transmitted via other animals (Table 2). Most of the patients listed in Table 2 were either young, old, pregnant or immunosuppressed. We assume that inclusion in one of these groups is a risk factor for developing clinical symptoms after infection with rodent-associated bartonellae because these groups are associated with an impaired or not yet fully developed immune system. Thus, rodent-associated bartonellosis seems to be opportunistic and might be more likely to develop when a person has a pre-existing medical condition. Furthermore, the studies showed that being homeless [24], abusing drugs [20], or being in contact with animals, e.g. through hunting or animal breeding, may increase the risk of rodent-associated *Bartonella* infection.

## Reservoir role and clinical cases of pet animals infected with rodent-associated *Bartonella* spp.

Cats are known hosts of *B. henselae* (and *Bartonella clarridgeiae* and *Bartonella kohlerae*) and dogs of *Bartonella rochalimae*. Thus far, there have only been occasional reports of clinical symptoms in cats and dogs related to *Bartonella* spp. infection, and even fewer related to rodent-associated bartonellae. Whether bartonellae are primary or opportunistic pathogens for cats and dogs is not entirely clear. Clinical manifestations of bartonellosis are rarely seen in domestic cats, and to the best of our knowledge, there have been no case reports of rodent-associated bartonellosis in them. However, there is one report of a cat which was thought to have transmitted rodent-associated *B. grahamii* to a human via a scratch [25]. Unlike cats, dogs may develop severe clinical symptoms of bartonellosis that are similar to those displayed by humans [26]. Thus far, rodent-associated *B. elizabethae, B. grahamii, B. taylori* and a *Bartonella volans*-like strain have been detected in dogs [27–29]. However, only *B. elizabethae* infections could be linked directly to a canine clinical case. An 8-year-old dog suffering from unspecific symptoms including lethargy, appetite and weight loss was diagnosed with *B. elizabethae* infection in the blood stream. The dog died immediately before the diagnosis was confirmed, and no other pathogen was detected in the blood [28]. Furthermore, there is one record of a dog with a previously unspecified clinical record which was found to be positive for a strain of *B. volans* after its death [29]. *Bartonella grahamii, B. elizabethae, B. taylorii* were found to have a moderate prevalence (9.4%) in stray dogs without a clinical record in Thailand, highlighting the potential reservoir competence of dogs for rodent-associated bartonellae [27].

## Current insights into *Bartonella* taxonomy with a focus on recently discovered small mammal-associated *Bartonella* spp.

 Bartonellae can be divided into eubartonellae and other ancient clades according to their genetic features [5]. Eubartonellae can further be subdivided into four lineages, one of which is the most diverse with regard to potential host species as well as species and subspecies of *Bartonella*. There are presently 84 known species and subspecies of *Bartonella*, of which 38 were initially found in specimens belonging to the order Rodentia, followed by 10 species each in specimens of the orders Carnivora and Artiodactyla, and seven in humans. The number of newly described species has been increasing in the past decade [5]. Forty-five new *Bartonella* species have been proposed and/or published since 2011 (Table 1). Most of these newly described *Bartonella* species (*n* = 30) were reported in wild small mammal or specifically rodent species such as *Acomys russatus* (golden spiny mouse), *Mastomys erythroleucus* (Guinea multimammate mouse), and *Pachyuromys duprasi* (fat-tailed gerbil) (Table 1). Out of the 51 proposed or published rodent- or other small mammal-associated species, 19 have *Candidatus* status.

There is an increasing number of newly discovered, not yet fully characterized, *Bartonella* species with *Candidatus* status. Labelling a potentially new species *Candidatus* is a new concept that began in the 1990s [30] and allows researchers to propose prokaryotic taxa that are well characterized but as yet uncultured. In contrast to official species names, there is no official process for reviewing proposed *Candidatus* species and their names before they are published (A. Oren, personal communication). Authors are welcome to submit the proposed names to the Judicial Commission on Prokaryote Nomenclature of the International Committee on Systematics of Prokaryotes (ICSP), but they are not obliged to do so. The committee reviews *Candidatus* species through an extensive literature review, as many *Candidatus* names have not been qualitatively validated or do not follow the rules of the International Code of Nomenclature of Prokaryotes [31], which explains why many *Candidatus* names in use for newly described *Bartonella* species are malformed. However, suggested corrections for these malformed names are regularly published [32]. Nevertheless, due to the large number of new *Candidatus* taxa being proposed, particularly those with rodents and other small mammals as their origin and likely reservoir hosts, the lists of corrected names are not exhaustive and constantly evolving [31, 32].

Thus, the above should be borne in mind with respect to newly described *Candidatus* species and their published names. A mandatory submission process for the validation of species names prior to the publication of newly proposed *Candidatus* species would help to avoid the time-consuming renaming process carried out by the ICSP, and would further help to avoid the circulation in the literature of malformed *Candidatus* names. For example, the ICSP proposes that ‘*Bartonella bandicootii*’ should be re-named ‘*Bartonella paramelis’* (Table 1). ‘Bandicoot’ is the English name of the animal from which this species of *Bartonella* was isolated, but the proposed specific epithet ‘bandicootii’ (rather than ‘bandicooti’) is both malformed and in violation of recommendation 6 (3) of the ICNP. The genus name of the host animal, which is *Parameles*, should be used as the basis for the specific epithet of the *Bartonella* species rather than the English common name, bandicoot, which is why the current name of this species should be *Bartonella paramelis* [33].

Furthermore, an increasing number of *Bartonella* species without *Candidatus* status have not been validly published according to the ICNP. In total, there are 44 *Bartonella* species names currently in use that have not been validly published. Of these species, 40 have been described since 2007, and 17 are from rodents or other small mammals (Table 1). As the number of newly described *Bartonella* species is increasing, in particular with respect to those isolated from rodents and other small mammals, it would be helpful if their names were proposed for approval under the ICNP before they were published, and that these should only be published when the relevant dataset allows these *Bartonella* species to be clearly differentiated from known species and subspecies of the genus. At present, it is not always easy to define a species or subspecies, but with whole genome sequencing becoming more affordable, it is highly likely that this issue will be resolved in the future through the sufficient description of new *Candidatus* species through the use of this genetic technique [34].

## Further reasons for low numbers of cases of bartonellosis in humans despite high prevalences of *Bartonella* spp. in rodents and other small mammals

At first glance, it appears to be a phenomenon that *Bartonella* spp. from small mammals seem to be the least pathogenic for humans and their companion animals, and especially so in the case of rodents, as these comprise the group of small mammals with the highest prevalences and diversities of recorded *Bartonella* species, are commonly found in urban areas, and are known to harbour and spread disease [35]. Recent work demonstrated that one third of Norway rats in the Belgian region of Flanders harboured *B. tribocorum*, yet no human cases of infection with this parasite have been reported there [36]. Although it does not seem plausible that there is no transmission of *Bartonella* spp. from rodents to humans, in particular when taking into consideration the prevalences of *Bartonella* spp. in rodents and the frequent proximity of the latter to humans, there are several factors that might account for this.

Rodent-associated *Bartonella* spp. are probably arthropod-borne pathogens, but transmission through rodent scratches or bites (as is often reported for *B. henselae* infections) cannot be completely ruled out [1, 37]. Several studies have reported possible vertical transmission in naturally infected rodents [37–39]. However, a study on infected *Clethrionomys glareolus* could not experimentally prove transplacental or transovarial transmission of *B. taylorii* and *B. grahamii* to the offspring. There is one report of possible vertical transmission of *B. birtlesii* in BALB/c mice [40]. Most rodent-associated flea species are host specific and do not infest humans, which further limits potential zoonotic transmission of *Bartonella* spp. However, a few flea species are known to be more host opportunistic [41]. *Yersinia pestis*, the causative agent of the plague, is known to be transmitted from rats to humans mainly through *Xenopsylla cheopis*, the oriental rat flea [42], which can cause severe outbreaks of the disease. *Xenopsylla cheopis* is also known to harbour DNA of zoonotic *Bartonella* spp. such as *B. elizabethae*, and it was experimentally shown that *X. cheopis* can excrete this species over time [43, 44]. Nonetheless, there are, to the best of our knowledge, no reported cases of bartonellosis in humans previously infested with this flea species.

Another possible means of infection is direct contact with a reservoir host. Cats may transmit *B. henselae* to humans through their scratches [45]. There is even one report of a cat transmitting *B. grahamii*, which is a rodent-associated pathogen, to its owner [25]. The explanation for this was that the cat came into contact with an infected rodent when it caught it with its paws, which then became infectious. As rodents are not predators of humans and tend to avoid them, direct contact between the two is highly unlikely, with the exception of small mammals kept as pets [46]. However, indirect transmission via a cat is a possible, though unlikely, transmission path [25].

Notwithstanding the cases described above, rodent-associated bartonellae are the principal cause of bartonellosis in humans. However, how and to what extent rodents and their ectoparasites are involved in the zoonotic transmission cycle of *Bartonella* is not fully understood. Studying the transmission of *Bartonella* spp. from rodents to humans would help in assessing the potential risk of the former for the latter.

## Conclusions

The zoonotic transmission cycle of rodent-associated bartonellae is not fully understood. It is especially unknown whether *Bartonella* infections in humans arise from direct contact with small mammals or rather indirectly via infestation with a rodent-associated ectoparasite vector. The total number of confirmed human cases of bartonellosis worldwide caused by rodent-associated bartonellae is much lower than expected when taking into account the abundance of *Bartonella* spp. in rodents, and possible reasons for this have been discussed in this review. Many small mammal species are considered reservoir hosts for the increasing number of newly described *Bartonella* spp., although some of the latter have yet to be experimentally confirmed. Even though most *Bartonella* spp. show high adaptation to their hosts, this is not the case for all rodent-associated species. That is why further experimental research is needed to increase our understanding of host–pathogen interactions between rodents and *Bartonella* species.

## Supplementary Information

**Additional file 1: Table S1.** Worldwide prevalence levels of *Bartonella* spp. in small mammal species including the detection method.

## Data Availability

Not applicable.

## Abbreviations

ICNP: International Code of Nomenclature of Prokaryotes
ICSP: International Committee on Systematics of Prokaryotes
Trw T4SS: Trw type IV secretion system
