# Supplementary material for: Emerging rodent-associated Bartonella: a threat for human health?
Source: Parasit Vectors. 2022 Mar 31;15:113. doi: 10.1186/s13071-022-05162-5 (PMC8969336; doi:10.1186/s13071-022-05162-5)
Supplement: Supplementary file 1 — Additional file 1: Table S1. Worldwide prevalence levels of Bartonella spp. in small mammal species including the detection method. [file 13071_2022_5162_MOESM1_ESM.docx]

Additional file 1: Table S1 Worldwide prevalence levels of *Bartonella* spp. in small mammal species including the detection method

| **Small mammal species** | **Small mammal family** | **Country** | ***Bartonella* prevalence** | **Sample size** | ***Bartonella* species** | **Detection method** | **Reference** |
| --- | --- | --- | --- | --- | --- | --- | --- |
| ***Acomys cahirinus*** | Muridae | Egypt | 9.7 % | 31 | *B. elizabethae*-like | culture/ PCR (*gltA*) | [47] |
|  |  | Israel | 9% | 57 | *Bartonella acomydis,* unknown *B.* spp. | culture/ PCR (*ssrA/ gltA/ rpoB/ 16S–23S rRNA*) | [48] |
|  |  |  | 25% | 4 | *B. elizabethae*-like | PCR (*16S-23S rRNA*/ *gltA*/ *rpoB*) | [49] |
| ***Acomys dimidiatus*** | Muridae | Egypt | 2.5% | 168 | n/a | blood smear | [50] |
| ***Acomys russatus*** | Muridae | Egypt | 61.5 % | 13 | *B. elizabethae*-like, unknown *B.* spp. | culture/ PCR (*gltA*) | [47] |
|  |  | Egypt, Thailand or the Netherlands | n/a | n/a | *B. acomydis* | PCR (*16S rRNA/ ftsZ/ gltA/ rpoB/ 16S–23S rRN*A) | [51] |
| ***Acomys wilsoni*** | Muridae | Tanzania | 0% | 6 | n/a | PCR (*ssrA/ gltA*) | [52] |
| ***Aethomys ineptus*** | Muridae | South Africa | 86.7% | 75 | *Bartonella queenslandensis-*like | PCR (*gltA/ ribC/ rpoB/ nuoG*) | [53] |
| ***Aethomys namaequensis*** | Muridae | South Africa | 57.9 % | 19 | *B. elizabethae, B. grahamii* | culture/ PCR (*gltA*) | [54] |
| ***Allactaga sibirica*** | Dipodidae | China | 20% | 5 | n/a | PCR (*gltA*) | [55] |
|  |  |  | 100% | 3 | *B. jaculi* | culture / PCR (*gltA/ ftsZ/ rpoB/ ribC*) | [56] |
| ***Anourosorex squamipes*** | Soricidae § | China | 0% | 2 | n/a | culture/ PCR (*gltA*) | [57] |
| ***Apodemus agrarius*** | Muridae | Austria | 12.5% | 32 | *B. taylorii,* unknown *B*. spp. | PCR (*16S-23S rRNA*) | [58] |
|  |  | Bulgaria | 9.7% | 31 | unknown *B.* spp. | blood smear | [59] |
|  |  | China | 47% | 192 | *B. fuyuanensis, B. grahamii* | PCR (*gltA*/ *rpoB*) | [60] |
|  |  |  | 11.1 % | 72 | *B. fuyuanensis, B. grahamii* | PCR (*gltA*/ *rpoB*) | [61] |
|  |  |  | 45.5% | 11 | *B. coopersplainsensis, B. grahamii, B. rattimassiliensis, B. taylorii* | PCR (*gltA/ 16S rRN/ ftsZ/ rpoB*) | [62] |
|  |  | Croatia | 13.2 % | 53 | unknown *B.* spp. | PCR (hbpA/ *gltA/ 16S rRNA*) | [63] |
|  |  | Germany | 0% | 3 | n/a | PCR (*16S-23S rRNA*) | [64] |
|  |  |  | 0% | 1 | n/a | PCR (*16S-23S rRNA*) | [65] |
|  |  |  | 50% | 6 | *B. taylorii* | PCR (*16S-23S rRNA/ gltA*) | [66] |
|  |  | Lithuania | 15.8 % | 76 | *B. coopersplainensis, B. grahamii, B. tribocorum* | PCR (*16S-23S rRNA*/ *rpoB*/ *groEL*/ *ssrA*) | [67] |
|  |  | Poland | 38.3 % | 94 | *B. birtlesii, B. elizabethae-*like*, B. grahamii, B. taylorii* | PCR (*gltA*/ *ribC*/ *groEL*/ *virB5*) | [68] |
|  |  | Russia | 73% | 15 | *B. grahamii, B. taylorii,* unknown *B.* spp. | PCR (*gltA*) | [69] |
|  |  |  | 33% | 33 | *B. grahamii* | culture/ PCR (*gltA/ rpoB*) | [70] |
|  |  | Slovakia | 0.8% | 370 | n/a | blood smear | [71] |
|  |  |  | 9% | 344 | *B. grahamii, B. taylorii, B. birtlesii, B. clarridgeiae/B. rochalimae* clade*, B. elizabethae/B. tribocorum* clade | PCR (*gltA/ rpoB/ groEL*) | [72] |
|  |  | Slovenia | 26.6 % | 30 | *B. grahamii,* unknown *B.* spp*.* | PCR (*16S-23S rRNA/ ftsZ*) | [73] |
|  |  | South Korea | 6.7 % | 358 | *B. elizabethae* | culture/ PCR (*gltA*) | [74] |
|  |  |  | 14.2 % | 373 | *B. birtlesii, B. doshiae, B. elizabethae, B. henselae* | PCR (*23S rRNA/ groEL*) | [75] |
|  |  |  | 58% | 24 | *B. grahamii, B. japonica,* unknown *B.* spp. | culture/ PCR (*gltA/ rpoB*) | [70] |
|  |  | Taiwan | 93% | 15 | *B. tribocorum,* unknown *B.* spp. | culture/ PCR (*gltA/ rpoB*) | [70] |
|  |  | Turkey | 5.5% | 18 | *B. taylorii* | PCR (*16S-23S rRNA/ rpoB/ gltA)* | [76] |
|  |  | Ukraine | 28.6 % | 7 | n/a | PCR (*rpoB*) | [77] |
| ***Apodemus argenteus*** | Muridae | Japan | 54.3 % | 35 | *B. elizabethae, B. grahamii, B. tribocorum,* unknown *B.* spp. | culture/ PCR (*gltA*/ *rpoB*) | [78] |
|  |  |  | n/a | 5 | *B. grahamii* | PCR (*16S rRNA/ ftsZ/ gltA/ groEL/ ribC/ rpoB*) | [79] |
|  |  |  | n/a | n/a | *B. japonica* | PCR (*16S rRNA/ ftsZ/ gltA/ groEL/ ribC/ rpoB/ 16S-23S rRNA*) | [80] |
|  |  |  | 60% | 5 | *B. japonica, B. grahamii* | culture/ PCR (*gltA*/ *rpoB*) | [81] |
|  |  |  | 69% | 29 | *B. grahamii, B. silvatica, B. phoceensis, B. japonica* | culture/ PCR (*gltA/ rpoB*) | [70] |
| ***Apodemus chevrieri*** | Muridae | China | 18.5% | 27 | n/a | PCR (*gltA*) | [55] |
|  |  |  | n/a | 1 | *B. grahamii* | PCR (*16S rRNA/ ftsZ/ gltA/ groEL/ ribC/ rpoB*) | [79] |
|  |  |  | 62.5 % | 84 | *B. grahamii*-like, unknown *B.* spp*.* | culture/ PCR (*gltA*) | [57] |
| ***Apodemus draco*** | Muridae | China | 18.5% | 27 | n/a | PCR (*gltA*) | [55] |
|  |  |  | n/a | 1 | *B. grahamii* | PCR (*16S rRNA/ ftsZ/ gltA/ groEL/ ribC/ rpoB*) | [79] |
|  |  |  | 33.3 % | 14 | *B. grahamii*-like, unknown *B.* spp*.* | culture/ PCR (*gltA*) | [57] |
| ***Apodemus flavicollis*** | Muridae | Austria | 10.3% | 29 | *B. taylorii* | PCR (*16S-23S rRNA*/ *rpoB)* | [82] |
|  |  | Bulgaria | 23.4% | 64 | unknown *B.* spp. | blood smear | [59] |
|  |  | Croatia | 12.2 % | 131 | *B. elizabethae, B. grahamii* | PCR (hbpA/ *gltA/ 16S rRNA*) | [63] |
|  |  | Denmark | 53.3 % | 15 | *B. grahamii, B. taylorii, B. tribocorum* | culture/ PCR (*16S rRNA*) | [83] |
|  |  | Germany | 84.4 % | 32 | *B. grahamii, B. taylorii,* unknown *B*. spp. | PCR (*16S-23S rRNA*) | [64] |
|  |  |  | 78% | 59 | *B. taylorii,* unknown *B*. spp. | PCR (*16S-23S rRNA*) | [65] |
|  |  |  | 73.3% | 172 | *B. taylorii, B. doshiae,* unknown B. spp. | PCR (*16S-23S rRNA/ gltA*) | [66] |
|  |  | Greece | 31.3 % | 61 | *B. grahamii, B. birtlesii, B. taylorii* | PCR (*gltA*) | [84] |
|  |  | Lithuania | 33.2% | n/a | *B. grahamii, B. taylorii* | PCR (*16S-23S rRNA*/ *gltA*) | [85] |
|  |  |  | 79.6 % | 201 | *B. grahamii, B. taylorii* | PCR (*16S-23S rRNA*/ *rpoB*/ *groEL*/ *ssrA*) | [67] |
|  |  | Poland | 48.2 % | 663 | *B. birtlesii, B. grahamii, B. taylorii* | PCR (*gltA*) | [86] |
|  |  |  | 42.2 % | 68 | *B. grahamii* | PCR (*gltA*) | [87] |
|  |  |  | 46.3 % | 162 | n/a | PCR (*gltA*) | [88] |
|  |  | Russia | n/a | 1 | *B. grahamii* | PCR (*16S rRNA/ ftsZ/ gltA/ groEL/ ribC/ rpoB*) | [79] |
|  |  | Slovakia | 63.0 % | 387 | *B. clarridgeiae*-like, *B. doshiae*-like*, B. elizabethae, B. grahamii, B. taylorii* | PCR (*16S-23S rRNA*) | [89] |
|  |  | Slovenia | 62.7 % | 43 | *B. taylorii,* unknown *B.* spp*.* | PCR (*16S-23S rRNA/ ftsZ*) | [73] |
|  |  | Spain | 66.7 % | 3 | *B. taylorii, B. vinsonii* subsp. *arupensis* | PCR (*16S-23S rRNA*/ *16S rRNA*/ *gltA*) | [90] |
|  |  | Sweden | 17.3 % | 110 | *B. grahamii, B. clarridgeiae, B. taylorii* | culture/ PCR (*gltA*) | [91] |
|  |  | Turkey | 85% | 47 | *B. taylorii, B. grahamii, B. birtlesii* | culture/ PCR *(gltA*) | [92] |
|  |  |  | 32.1% | 56 | *B. taylorii* | PCR (*16S-23S rRNA/ rpoB/ gltA)* | [76] |
|  |  | Ukraine | 75% | 12 | *B. grahamii, B. taylorii* | PCR (*rpoB*) | [77] |
| ***Apodemus latronum*** | Muridae | China | n/a | 2 | *B. grahamii* | PCR (*16S rRNA/ ftsZ/ gltA/ groEL/ ribC/ rpoB*) | [79] |
|  |  |  | 71.4 % | 19 | *B. grahamii*-like, unknown *B.* spp. | culture/ PCR (*gltA*) | [57] |
| ***Apodemus mystacinus*** | Muridae | Turkey | 0% | 7 | n/a | culture/ PCR *(gltA*) | [92] |
| ***Apodemus peninsulae*** | Muridae | China | 17.7% | 17 | n/a | PCR (*gltA*) | [55] |
|  |  |  | 28.6 % | 7 | *B. grahamii* | PCR (*gltA*) | [60] |
|  |  |  | 50% | 6 | *B. taylorii, B. japonica* | PCR (*gltA/ 16S rRNA/ ftsZ/ rpoB*) | [62] |
|  |  | Russia | 60% | 15 | *B. grahamii, B. taylorii,* unknown *B.* spp. | PCR (*gltA*) | [69] |
|  |  |  | 54% | 13 | *B. grahamii, B. taylorii* | culture/ PCR (*gltA/ rpoB*) | [70] |
|  |  | South Korea | 0% | 3 | n/a | PCR (*23S rRNA*/ *groEL*) | [75] |
| ***Apodemus* sp.** | Muridae | Switzerland | 31.8 % | 129 | *B. birtlesii, B. grahamii, B. taylorii* | PCR (*gltA*/ *rpoB/ 16S-23S rRNA*) | [93] |
| ***Apodemus speciosus*** | Muridae | Japan | 60.3 % | 224 | *B. elizabethae, B. grahamii, B. taylorii, B. tribocorum, unknown B. spp.* | culture/ PCR (*gltA*/ *rpoB*) | [78] |
|  |  |  | n/a | 10 | *B. grahamii* | PCR (*16S rRNA/ ftsZ/ gltA/ groEL/ ribC/ rpoB*) | [79] |
|  |  |  | n/a | n/a | *B. silvatica* | PCR (*16S rRNA/ ftsZ/ gltA/ groEL/ ribC/ rpoB/ 16S-23S rRNA*) | [80] |
|  |  |  | 93.6 % | 31 | *B. grahamii, B. japonica* | PCR (*gltA*/ *rpoB*) | [81] |
|  |  |  | 62% | 245 | *B. grahamii, B. japonica, B.silvatica, B. phoceenis, B. taylorii, B. queenslandensis* | culture/ PCR (*gltA/ rpoB*) | [70] |
| ***Apodemus sylvaticus*** | Muridae | Austria | 23.1% | 26 | *B. birtlesii, B. grahamii, B. taylorii* | PCR (*16S-23S rRNA*/ *rpoB)* | [82] |
|  |  | Croatia | 0% | 3 | n/a | PCR (hbpA/ *gltA/ 16S rRNA*) | [63] |
|  |  | Denmark | 53.3 % | 15 | *B. henselae, B. taylorii, B. vinsonii* subsp*. vinsonii* | culture/ PCR (*16S rRNA*) | [83] |
|  |  | France | 12.8 % | 70 | *B. taylorii* | PCR (*gltA*/ *ftsZ*/ *groEL*/ *rpoB*/ *ribC*/ *nuoG*/ *virB5*) | [94] |
|  |  | Germany | 41.2% | 35 | *B. taylorii,* unknown *B*. spp. | PCR (*16S-23S rRNA/ gltA*) | [66] |
|  |  | Ireland | 30% | 288 | *B. birtlesii, B. taylorii* | PCR (*16S rRNA/ gltA)* | [95] |
|  |  | Israel | 0% | 1 | n/a | PCR (*16S-23S rRNA*/ *gltA*/ *rpoB*) | [49] |
|  |  | Slovakia | 0% | 2 | n/a | PCR (*16S-23S rRNA*) | [89] |
|  |  | Slovenia | 34.4 % | 32 | *B. taylorii* | PCR (*16S-23S rRNA/ ftsZ*) | [73] |
|  |  | Spain | 31.4 % | 220 | *B. birtlesii, B. doshiae, B. elizabethae, B. grahamii, B. rochalimae, B. taylorii, B. vinsonii* subsp. *arupensis* | PCR (*16S-23S rRNA*/ *16S rRNA*/ *gltA*) | [90] |
|  |  | Sweden | 24% | 25 | *B. birtlesii, B. grahamii* | culture/ PCR (*gltA*) | [91] |
|  |  | UK | n/a | n/a | *B. doshiae, B. grahamii, B. taylorii* | PCR (*gltA*) | [96] |
|  |  |  | 7.4-37.5 | 405 | *B. grahamii, B. birtlesii, B. taylorii, B. doshiae,* unknown *B.* spp. | PCR | [97] |
|  |  |  | 59.3% | 743 | *B. grahamii, B. taylorii, B. birtlesii, B. doshiae*-like, unknown *B.* spp. | PCR (*16S-23S rRNA*) | [98] |
|  |  | Ukraine | 0% | 1 | n/a | PCR (*rpoB*) | [77] |
| ***Apodemus uralensis*** | Muridae | Georgia | 25% | 8 | *B. elizabethae, B. tribocorum* | culture/ PCR (*gltA*) | [99] |
|  |  | Russia | n/a | 1 | *B. grahamii* | PCR (*16S rRNA/ ftsZ/ gltA/ groEL/ ribC/ rpoB*) | [79] |
|  |  | Turkey | 88% | 17 | *B. taylorii, B. birtlesii* | culture/ PCR *(gltA*) | [92] |
| ***Apodemus witherbyi*** | Muridae | Turkey | 72% | 44 | *B. taylorii, B. grahamii, B. birtlesii* | culture/ PCR *(gltA*) | [92] |
| ***Arvicanthis dembeensis*** | Muridae | Ethiopia | 37.9 % | 224 | *B. birtlesii, B. elizabethae,* unknown *B.* spp*.* | PCR (*rpoB*) | [100] |
| ***Arvicanthis neumanni*** | Muridae | Congo | 40% | 5 | *B. elizabethae, B. grahamii* | PCR (*gltA*/ *rpoB*) | [101] |
| ***Arvicanthis niloticus*** | Muridae | Uganda | 35.5% | 31 | unknown *B.* spp. | PCR (*16S-23S rRNA*) | [102] |
| ***Arvicola amphibius*** | Cricetidae | Belgium | 100% | 2 | *B. doshiae,* unknown *B.* spp. | PCR (*16S-23S rRNA*) | [36] |
|  |  | UK | 26.7% | 75 | n/a | PCR | [103] |
| ***Arvicola sherman*** | Cricetidae | Switzerland | 10.5% | 86 | *B. doshiae, B. taylorii* | PCR (*gltA/ rpoB/ 16S-23S rRNA*) | [93] |
| ***Arvicola terrestris*** | Cricetidae | Germany | 100% | 3 | *B. grahamii,* unknown *B*. spp. | PCR (*16S-23S rRNA*) | [64] |
|  |  |  | 0% | 1 | n/a | PCR (*16S-23S rRNA*) | [65] |
| ***Atelerix algirus*** | Erinaceidae § | Algeria | 75% | 46 | *B. elizabethae, B. tribocorum* | PCR (*16S-23S rRNA*/ *ftsZ*) | [104] |
| ***Bandicota bengalensis*** | Muridae | Bangladesh | 63.2% | 76 | *B. elizabethae, B. tribocorum* | culture/ PCR (*gltA*) | [105] |
|  |  | Myanmar | 61.9 % | 155 | *B. kosoyi,* unknown *B.* spp. | PCR (*nuoG/ gltA/ 16S-23S rRNA*) | [106] |
|  |  | Nepal | 26.3 % | 38 | *B. coopersplainensis, B. queenslandensis, B. rochalimae* | PCR (*gltA*/ *rpoB*) | [107] |
|  |  | Sri Lanka | 100% | 1 | unknown *B.* spp. | PCR (*nuoG/ gltA/ 16S-23S rRNA*) | [106] |
| ***Bandicota indica*** | Muridae | Cambodia | 0% | 3 | n/a | culture/ PCR (*gltA/ 16S-23S rRNA*) | [108] |
|  |  | Laos | 0% | 2 | n/a | PCR (*16S-23S rRNA*/ *gltA*/ *rpoB*) | [109] |
|  |  |  | 0% | 6 | n/a | culture/ PCR (*gltA/ 16S-23S rRNA*) | [108] |
|  |  | Myanmar | 75% | 8 | *B. kosoyi* | PCR (*nuoG/ gltA/ 16S-23S rRNA*) | [106] |
|  |  | Sri Lanka | 0% | 13 | n/a | PCR (*nuoG/ gltA/ 16S-23S rRNA*) | [106] |
|  |  | Taiwan | 100% | 2 | *B. tribocorum* | culture/ PCR (*gltA/ rpoB*) | [70] |
|  |  | Thailand | 32.6 % | 46 | *B. coopersplainensis, B. rattimassiliensis* | culture/ PCR (*gltA*) | [110] |
|  |  |  | 8.1 % | 147 | *B. elizabethae-*like*,* unknown *B.* spp. | culture/ PCR (*gltA*) | [111] |
|  |  |  | 7.7 % | 65 | *B. elizabethae, B. queenslandensis, B. tribocorum* | culture/ PCR (*gltA/ 16S-23S rRNA*) | [108] |
|  |  |  | 15.1% | 279 | *B. coopersplainensis* | culture/ PCR *(gltA/* ssrA) | [112] |
|  |  |  | 10.7% | 28 | *B. tribocorum, B. rattimassiliensis* | culture/ PCR *(gltA/* *rpoB*) | [113] |
|  |  |  | 0% | 20 | n/a | PCR (*23S rRNA/ gltA/ ftsZ/ 16S-23S rRNA/ rpoB*) | [114] |
| ***Bandicota savilei*** | Muridae | Cambodia | 9.5 % | 74 | *B. coopersplainensis, B. queenslandensis, B. rattimassiliensis* | culture/ PCR (*16S-23S rRNA*/ *gltA*) | [108] |
|  |  | Laos | 0% | 1 | n/a | PCR (*16S-23S rRNA*/ *gltA*/ *rpoB*) | [115] |
|  |  |  | 7.1 % | 14 | *B. coopersplainensis, B. queenslandensis, B. rattimassiliensis* | culture/ PCR (*16S-23S rRNA*/ *gltA*) | [108] |
|  |  | Thailand | 57.1 % | 7 | *B. rattimassiliensis, B. tribocorum* | culture/ PCR ( *gltA*) | [110] |
|  |  |  | 0% | 24 | n/a | culture/ PCR (*gltA/ 16S-23S rRNA*) | [108] |
|  |  |  | 35.7% | 14 | n/a | culture/ PCR *(gltA/* ssrA) | [112] |
|  |  |  | 6.1 % | 33 | *B. queenslandensis, B. rochalimae* | PCR (*23S rRNA*/ *gltA*/ *ftsZ*/ *16S-23S rRNA*/ *rpoB*) | [114] |
| ***Bandicota* sp.** | Muridae | Laos | 0% | 1 | n/a | culture/ PCR (*16S-23S rRNA*/ *gltA*) | [108] |
| ***Berylmys berdmorei*** | Muridae | Cambodia | 9.1 % | 11 | *B. queenslandensis* | culture/ PCR (*16S-23S rRNA*/ *gltA*) | [108] |
|  |  | Laos | 20% | 5 | *B. queenslandensis* | culture/ PCR (*16S-23S rRNA*/ *gltA*) | [108] |
|  |  | Thailand | 100% | 1 | *B. tribocorum* | culture/ PCR (*gltA*) | [110] |
|  |  |  | 13.3 % | 15 | *B. queenslandensis* | culture/ PCR (*16S-23S rRNA*/ *gltA*) | [108] |
| ***Berylmys bowersi*** | Muridae | Laos | 0% | 2 | n/a | culture/ PCR (*16S-23S rRNA*/ *gltA*) | [108] |
|  |  | Thailand | 0% | 15 | n/a | culture/ PCR (*16S-23S rRNA*/ *gltA*) | [108] |
| ***Blarina brevicauda*** | Soricidae § | USA | 25% | 4 | n/a | culture/ PCR (*gltA*)/ IFA | [116] |
| ***Callosciurus erythraeus*** | Sciuridae | Laos | 0% | 2 | n/a | PCR (*16S-23S rRNA*/ *gltA*/ *rpoB*) | [109] |
| ***Callosciurus notatus*** | Sciuridae | Egypt, Thailand or the Netherlands | n/a | n/a | *B. callosciuri* | PCR (*16S rRNA/ ftsZ/ gltA/ rpoB/ 16S–23S rRN*A) | [51] |
|  |  | Thailand | 63.3 % | 30 | *B. elizabethae*-like, unknown *B.* spp. | culture/ PCR (*gltA*) | [47] |
| ***Cannomys badius*** | Spalacidae | Laos | 0-50 % | 2 | Lao/Nh2 | PCR (*16S-23S rRNA*/ *gltA*/ *rpoB*) | [109] |
|  |  |  | 0% | 1 | Lao/Nh2 | culture/ PCR (*16S-23S rRNA*/ *gltA*/ *rpoB*) | [108] |
| ***Cavia porcellus*** | Caviidae | Peru | 19.3% | 83 | *B. rochalimae* | PCR (*16S-23S rRNA/ gltA*) | [117] |
| ***Chaetodipus hispidus*** | Heteromyidae | Mexico | 14% | 7 | unknow*n B*. spp. | PCR (*gltA*) | [118] |
|  |  | USA | 0% | 31 | n/a | culture/ PCR (*gltA*) | [119] |
| ***Chaetodipus penicullatus*** | Heteromyidae | Mexico | 12.9% | 31 | unknow*n B*. spp. | PCR (*gltA*) | [118] |
| ***Cricetomys gambianus*** | Nesomyidae | Nigeria | 1.1 % | 177 | *B. elizabethae* | PCR (*gltA*) | [120] |
|  |  | Uganda | 60% | 5 | *B. elizabethae,* unknown *B.* spp. | PCR (*16S-23S rRNA*) | [121] |
| ***Cricetomys* sp.** | Nesomyidae | Gabon | 67% | 3 | *B. massiliensis* | PCR (*16S-23S rRNA*) | [122] |
| ***Cricetulus barabensis*** | Cricetidae | China | 25% | 4 | n/a | PCR (*gltA*) | [55] |
|  |  |  | 0% | 8 | n/a | PCR (*gltA*/ *rpoB*) | [61] |
| ***Cricetulus longicaudatus*** | Cricetidae | China | 63% | 46 | *B. grahamii* | culture / PCR (*gltA/ ftsZ/ rpoB/ ribC*) | [56] |
| ***Cricetulus migratorius*** | Cricetidae | China | 0% | 2 | n/a | culture / PCR (*gltA/ ftsZ/ rpoB/ ribC*) | [56] |
| ***Cricetulus triton nestor*** | Cricetidae | South Korea | 0% | 1 | n/a | PCR (*23S rRNA*/ *groEL*) | [75] |
| ***Crocidura* cf. o*livieri*** | Muridae | Mali | 20% | 5 | unknown *B.* spp. | PCR (*gltA/ rpoB/ ftsZ/ 16S-23S rRNA*) | [123] |
| ***Crocidura dsinezumi*** | Soricidae § | South Korea | 0% | 2 | n/a | culture/ PCR (*gltA/ rpoB*) | [70] |
| ***Crocidura lasiura*** | Soricidae § | South Korea | 0% | 34 | n/a | culture/ PCR (*gltA*) | [74] |
|  |  |  | 12.1 % | 33 | n/a | PCR (*23S rRNA*/ *groEL*) | [75] |
| ***Crocidura olivieri*** | Soricidae § | Benin | 9.1% | 11 | unknown *B.* spp. | PCR (*rpoB/ gltA*) | [124] |
|  |  | Ethiopia | 2.4 % | 42 | n/a | PCR (*rpoB*) | [100] |
|  |  | Kenya | 7% | 16 | *B. birtlesii*-like | culture/ PCR (*gltA*) | [125] |
| ***Crocidura russula*** | Soricidae § | Germany | 0% | 3 | n/a | PCR (*16S-23S rRNA*) | [64] |
|  |  | Spain | 14.3 % | 8 | *B. birtlesii, B. tribocorum* | PCR (*16S-23S rRNA*/ *16S rRNA*/ *gltA*) | [90] |
| ***Crocidura shantungensis*** | Soricidae § | Taiwan | 100% | 1 | unknown *B.* spp. | culture/ PCR (*gltA/ rpoB*) | [75] |
| ***Crocidura* sp.** | Soricidae § | Benin | 0% | 2 | n/a | PCR (*rpoB/ gltA*) | [124] |
|  |  | Congo | 0% | 8 | n/a | PCR (*gltA*/ *rpoB*) | [101] |
|  |  | Laos | 0% | 1 | n/a | culture/ PCR (*16S-23S rRNA*/ *gltA*) | [108] |
| ***Crocidura suaveolens*** | Soricidae § | Turkey | 5% | 2 | unknown *B.* spp. | culture/ PCR *(gltA*) | [92] |
| ***Cynomys ludovicianus*** | Sciuridae | Mexico | 17.6% | 51 | unknow*n B*. spp. | PCR (*gltA*) | [118] |
|  |  | USA | 23.1 % | 1362 | n/a | culture/ PCR (*gltA*) | [126] |
| ***Deltamys kempi*** | Cricetidae | Argentina | 9.1 % | n/a | *B. grahamii, B. tribocorum,* unknown *B.* spp. | PCR (*16S-23S rRNA*/ *gltA*) | [127] |
| ***Dipodomys ordii*** | Heteromyidae | Mexico | 40% | 5 | n/a | PCR (gltA) | [118] |
|  |  | USA | 50% | 32 | unknown *B*. spp. | culture/ PCR (*gltA*) | [119] |
|  |  |  | 69.8% | 43 | *B. grahamii, B. doshiae, B. Bacilliformis-like* | PCR (*ssrA/ 16S-23S rRNA/ gltA*) | [128] |
|  |  |  | 0% | 3 | n/a | culture/ PCR (*16S rRNA*/ *gltA*/ *groEL*) | [129] |
| ***Dipodomys spectabilis*** | Heteromyidae | Mexico | 51.3% | 80 | unknow*n B*. spp. | PCR (*gltA*) | [118] |
|  |  | USA | 0% | 2 | unknow*n B*. spp. | PCR (*ssrA/ 16S-23S rRNA/ gltA*) | [128] |
| ***Dipomys merriami*** | Heteromyidae | Mexico | 57.1% | 189 | n/a | PCR (gltA) | [118] |
| ***Dremomys rufigenis*** | Sciuridae | Laos | 0% | 1 | n/a | PCR (*16S-23S rRNA*/ *gltA*/ *rpoB*) | [109] |
| ***Dryomys nitedula*** | Gliridae | Greece | n/a | n/a | *B. grahamii* | PCR (*gltA*) | [84] |
| ***Eothenomys melanogaster*** | Cricetidae | China | 33.3% | 3 | n/a | PCR (*gltA*) | [55] |
|  |  |  | 24% | 37 | *B. grahamii* | PCR (*gltA*) | [60] |
| ***Eothenomys miletus*** | Cricetidae | China | 18.8 % | 35 | unknown *B.* spp*.* | culture/ PCR (*gltA*) | [57] |
| ***Eothenomys regulus*** | Cricetidae | South Korea | 11.1 % | 9 | n/a | PCR (*23S rRNA*/ *groEL*) | [75] |
| ***Erinaceus concolor*** | Erinaceidae § | Israel | 33% | 3 | unknow*n B*. spp. | culture/ PCR (*ssrA/ gltA/ rpoB/ 16S–23S rRN*A) | [48] |
| ***Eutamias sibiricus*** | Sciuridae | China | 100% | 1 | *B. washoensis* subsp. *cynomysii* | PCR (*gltA/ 16S rRN/ ftsZ/ rpoB*) | [62] |
| ***Gerbillus andresoni*** | Muridae | USA | 35.7-75% | 16 | unknown *B.* spp*.* | PCR (*gltA*) | [130] |
| ***Gerbillus gambianus*** | Muridae | Senegal | n/a | 56 | *Candidatus* B. saheliensis | PCR (*gltA/ rpoB/ 16S rRNA/ ftsZ/ 16S-23S rRNA*) | [131] |
| ***Gerbillus nanus*** | Muridae | Saudi Arabia | 68.1 % | 138 | *B. elizabethae*-like, unknown *B.* spp. | PCR (*gltA/ nuoG*) | [132] |
| ***Gerbillus pyramidum*** | Muridae | Egypt | 90% | 10 | *B. elizabethae*-like, unknown *B.* spp. | culture/ PCR (*gltA*) | [47] |
| ***Glaucomys volans*** | Sciuridae | USA | 60% | 10 | *B. washoensis*-like | culture/ PCR (*gltA*) | [47] |
| ***Grammomys*sp.** | Muridae | Tanzania | 100% | 1 | *B. grahamii* | PCR (*gltA*/ *rpoB*) | [101] |
| ***Hemiechinus auritus*** | Erinaceidae § | Egypt | 0% | 10 | n/a | culture/ PCR (*gltA*) | [47] |
| ***Jaculus jaculus*** | Dipodidae | Egypt | 75% | 8 | *B. elizabethae*-like | culture/ PCR (*gltA*) | [47] |
| ***Jaculus orientalis*** | Dipodidae | Egypt | 81.3 % | 16 | *B. elizabethae*-like, unknown *B.* spp. | culture/ PCR (*gltA*) | [47] |
|  |  | Egypt, Thailand or the Netherlands | n/a | n/a | *B. jaculi* | PCR (*16S rRNA/ ftsZ/ gltA/ rpoB/ 16S–23S rRN*A) | [51] |
| ***Lemmiscus curtatus*** | Cricetidae | USA | 0% | 2 | n/a | culture/ PCR (*gltA*) | [119] |
| ***Lemniscomys barbarus*** | Muridae | Egypt | 0% | 11 | n/a | culture/ PCR (*gltA*) | [47] |
| ***Lemniscomys striatus*** | Muridae | Gabon | 0% | 27 | n/a | PCR (*16S-23S rRNA*) | [122] |
|  |  | Kenya | 50% | 2 | *B. tribocorum* | culture/ PCR (*gltA*) | [125] |
| ***Leopoldamys edwardsi*** | Muridae | Cambodia | 0% | 2 | n/a | culture/ PCR (*16S-23S rRNA*/ *gltA*) | [108] |
|  |  | Laos | 0% | 1 | n/a | culture/ PCR (*16S-23S rRNA*/ *gltA*) | [108] |
| ***Lepus europaeus*** | Leporidae # | Italy | 0% | 51 | n/a | PCR (*16S rRNA*) | [133] |
| ***Lophuromys rita*** | Muridae | Congo | 25% | 4 | *B. tribocorum* | PCR (*gltA*/ *rpoB*) | [101] |
| ***Lophuromys* sp.** | Muridae | Gabon | 3.7-33.9 % | 27 | *B. massiliensis, Candidatus* B. gabonensis | PCR (*16S-23S rRNA*) | [122] |
|  |  | Tanzania | 50% | 18 | *B. tribocorum, B. grahamii, B. birtlesii* | PCR (*gltA*/ *rpoB*) | [101] |
| ***Malacothrix typica*** | Nesomyidae | South Africa | 0% | 1 | n/a | culture/ PCR (*gltA*) | [54] |
| ***Mastomys awashensis*** | Muridae | Ethiopia | 37.5 % | 48 | *B. birtlesii, B. elizabethae* | PCR (*rpoB*) | [100] |
| ***Mastomys coucha*** | Muridae | Congo | 0% | 10 | n/a | PCR (*gltA*/ *rpoB*) | [101] |
| ***Mastomys erythroleucus*** | Muridae | Mali | 9.5% | 116 | *B. mastomydis, B. florencae,* unknown *B.* spp. | PCR (*gltA/ rpoB/ ftsZ/ 16S-23S rRNA*) | [123] |
|  |  | Senegal | 5-90.5% | n/a | n/a | PCR (*16S rRNA*) | [134] |
|  |  |  | n/a | 49 | *Candidatus* B. raoultii*, Candidatus B. mastomydis* | PCR (*gltA/ rpoB/ 16S rRNA/ ftsZ/ 16S-23S rRNA*) | [131] |
| ***Mastomys natalensis*** | Muridae | Kenya | 43% | 14 | *B. elizabethae, B. tribocorum* | culture/ PCR (*gltA*) | [125] |
|  |  | Mali | 23.1% | 26 | *B. mastomydis, B. florencae,* unknown *B.* spp. | PCR (*gltA/ rpoB/ ftsZ/ 16S-23S rRNA*) | [123] |
|  |  | Senegal | 59.1-95.8% | n/a | n/a | PCR (*16S rRNA*) | [134] |
|  |  | South Africa | 60% | 15 | unknown *B.* spp*.* | culture/ PCR (*gltA*) | [54] |
|  |  | Tanzania | 25% | 8 | *B. elizabethae* | PCR (*ssrA/ gltA*) | [52] |
| ***Mastomys* sp.** | Muridae | Benin | 25.9% | 27 | *B. tribocorum, B. elizabethae, B. taylorii, B. birtlesii* | PCR (*rpoB/ gltA*) | [124] |
| ***Maxomys ochraceiventer*** | Muridae | Malaysia | 0% | 2 | n/a | PCR (*gltA*) | [135] |
| ***Maxomys surifer*** | Muridae | Cambodia | 1.1 % | 92 | *B. queenslandensis* | culture/ PCR (*16S-23S rRNA*/ *gltA*) | [108] |
|  |  | Laos | 0% | 3 | n/a | culture/ PCR (*16S-23S rRNA*/ *gltA*) | [108] |
|  |  | Thailand | 0% | 6 | n/a | culture/ PCR (*16S-23S rRNA*/ *gltA*) | [108] |
| ***Maxomys whiteheadi*** | Muridae | Malaysia | 8.3% | 12 | n/a | PCR (*gltA*) | [135] |
| ***Melomys* sp*.*** | Muridae | Australia | n/a | 2 | *B. coopersplainensis, B. queenslandensis, B. rattaustraliani* | PCR (*gltA*/ *16S rRNA*/ *ftsZ*/ *16S–23S rRNA*) | [136] |
| ***Menetes berdmorei*** | Sciuridae | Thailand | 0% | 3 | n/a | culture/ PCR (*gltA*) | [111] |
| ***Meriones libycus*** | Muridae | Georgia | 42.6 % | 54 | unknown *B.* spp*.* | culture/ PCR (*gltA*) | [99] |
| ***Meriones meridianus*** | Muridae | China | 40% | 5 | n/a | PCR (*gltA*) | [55] |
|  |  |  | 54.6% | 11 | *B. krasnovii, Candidatus* B. gerbillinarum | culture / PCR (*gltA/ ftsZ/ rpoB/ ribC*) | [56] |
| ***Meriones shawii*** | Muridae | Algeria | 25% | 12 | unknown *B.* spp*.* | PCR (*16S-23S rRNA*/ *ftsZ*) | [104] |
| ***Meriones tristrami*** | Muridae | Egypt | 0% | 4 | n/a | culture/ PCR (*gltA*) | [47] |
|  |  | Israel | 58% | 43 | *Bartonella sp. Strain Mt-2286.1/Mt-2286.3* | culture/ PCR (*ssrA/ gltA/ rpoB/ 16S–23S rRN*A) | [48] |
| ***Meriones unguiculatus*** | Muridae | China | 34.7% | 216 | *B. elizabethae* | PCR (*gltA*) | [55] |
| ***Micaelamys namaquensis*** | Muridae | South Africa | 44% | 100 | *B. elizabethae* | PCR (*gltA*/ *rpoB*/ *nuoG*) | [53] |
| ***Micromys minutus*** | Muridae | Denmark | 0% | 15 | n/a | culture/ PCR (*16S rRNA*) | [83] |
|  |  | Lithuania | 23.7% | n/a | *B. taylorii* | PCR (*16S-23S rRNA*/ *gltA*) | [85] |
|  |  |  | 57.5 % | 40 | *B. grahamii* | PCR (*16S-23S rRNA*/ *rpoB*/ *groEL*/ *ssrA*) | [67] |
|  |  | Russia | 50% | 2 | *B. japonica* | culture/ PCR (*gltA/ rpoB*) | [70] |
|  |  | Slovakia | 0% | 1 | n/a | PCR (*16S-23S rRNA*) | [89] |
| ***Microtus agrestis*** | Cricetidae | Croatia | 0% | 1 | n/a | PCR (hbpA/ *gltA/ 16S rRNA*) | [63] |
|  |  | Denmark | 33.3 % | 15 | *B. grahamii, B. taylorii, B. vinsonii* subsp*. vinsonii* | culture/ PCR (*16S rRNA*) | [83] |
|  |  | France | 11% | 9 | *B. taylorii* | PCR (*gltA*/ *ftsZ*/ *groEL*/ *rpoB*/ *ribC*/ *nuoG*/ *virB5*) | [94] |
|  |  | Germany | 0% | 1 | n/a | PCR (*16S-23S rRNA/ gltA*) | [66] |
|  |  | Lithuania | 80% | 80 | *B. doshiae, B. grahamii, B. taylorii* | PCR (*16S-23S rRNA*/ *rpoB*/ *groEL*/ *ssrA*) | [67] |
|  |  | Poland | 59.2% | 17 | *B. taylorii* | culture/ PCR (*rpoB*) | [39] |
|  |  | Sweden | 33.3 % | 3 | *B. grahamii* | culture/ PCR (*gltA*) | [91] |
|  |  | Switzerland | 50% | 2 | *B. doshiae* | PCR (*gltA*/ *rpoB/ 16S-23S rRNA*) | [93] |
|  |  | UK | 34-57.5 % | 1533 | *B. doshiae, B. grahamii, B. taylorii* | culture/ PCR | [97] |
| ***Microtus arvalis*** | Cricetidae | Austria | 13.3% | 15 | *B. taylorii* | PCR (*16S-23S rRNA*/ *rpoB)* | [82] |
|  |  | Bulgaria | 28.6% | 7 | unknown *B.* spp. | blood smear | [59] |
|  |  | Croatia | 0% | 4 | n/a | PCR (hbpA/ *gltA/ 16S rRNA*) | [63] |
|  |  | Czech Republic | 77.6% | 147 | *B. doshiae, B. grahamii, B. taylorii,* unknown *B.* spp. | PCR (*16S-23S rRNA*) | [137] |
|  |  | Germany | 0% | 6 | n/a | PCR (*16S-23S rRNA/ gltA*) | [66] |
|  |  | Lithuania | 50% | 2 | *B. taylorii* | PCR (*16S-23S rRNA*/ *gltA*) | [85] |
|  |  |  | 33.3 % | 3 | *B. grahamii* | PCR (*16S-23S rRNA*/ *rpoB*/ *groEL*/ *ssrA*) | [67] |
|  |  | Poland | 72.6% | 124 | *B. taylorii, B. grahamii, B. doshiae, B. rochalimae-*like | culture/ PCR (*rpoB*) | [39] |
|  |  |  | 32.9 % | 50 | *B. grahamii* | PCR (*gltA*) | [87] |
|  |  |  | 36.9 % | 149 | n/a | PCR (*gltA*) | [88] |
|  |  | Slovakia | 61.1 % | 18 | *B. taylorii* | PCR (*16S-23S rRNA*) | [89] |
|  |  | Ukraine | 33.3 % | 3 | n/a | PCR (*rpoB*) | [77] |
| ***Microtus duodecimcostatus*** | Cricetidae | Spain | 0% | 1 | n/a | PCR (*16S-23S rRNA*/ *16S rRNA*/ *gltA*) | [90] |
| ***Microtus fortis*** | Cricetidae | China | 22% | 9 | *B. grahamii* | PCR (*gltA*) | [60] |
|  |  |  | 20% | 5 | *B. grahamii* | PCR (*gltA/ 16S rRN/ ftsZ/ rpoB*) | [62] |
|  |  | Russia | 83% | 6 | *B. taylorii* | PCR (*gltA*) | [69] |
| ***Microtus gapperi*** | Cricetidae | Canada | n/a | 6 | *B. grahamii* | PCR (*16S rRNA/ ftsZ/ gltA/ groEL/ ribC/ rpoB*) | [79] |
| ***Microtus hartingi*** | Cricetidae | Turkey | 6.25% | 16 | *B. taylorii* | PCR (*16S-23S rRNA/ rpoB/ gltA)* | [76] |
| ***Microtus maximowiczii*** | Cricetidae | China | 100% | 1 | *B. washoensis* subsp. *cynomysii* | PCR (*gltA/ 16S rRN/ ftsZ/ rpoB*) | [62] |
| ***Microtus montebelli*** | Cricetidae | Japan | 40% | 5 | *B. taylorii, unknown* B. *spp.* | culture/ PCR (*gltA/ rpoB*) | [70] |
| ***Microtus ochrogaster*** | Cricetidae | USA | 50% | 13 | unknown *B*. spp. | culture/ PCR (*gltA*) | [119] |
|  |  |  | n/a | 3 | *B. grahamii* | PCR (*16S rRNA/ ftsZ/ gltA/ groEL/ ribC/ rpoB*) | [79] |
| ***Microtus oeconomus*** | Cricetidae | China | 100% | 1 | *B. grahamii* | culture / PCR (*gltA/ ftsZ/ rpoB/ ribC*) | [56] |
|  |  | Lithuania | 12.5% | n/a | *B. grahamii* | PCR (*16S-23S rRNA*/ *gltA*) | [85] |
|  |  |  | 53.4 % | 58 | *B. grahamii, B. rochalimae, B. taylorii* | PCR (*16S-23S rRNA*/ *rpoB*/ *groEL*/ *ssrA*) | [67] |
|  |  | Poland | 58.8% | 76 | *B. taylorii, B. grahamii, B. doshiae, B. rochalimae-like* | culture/ PCR (*rpoB*) | [39] |
|  |  |  | 11.1 % | 14 | *B. grahamii* | PCR (*gltA*) | [87] |
|  |  |  | 13.7 % | 131 | n/a | PCR (*gltA*) | [88] |
| ***Microtus pinetorum*** | Cricetidae | USA | 33.3 % | 2 | n/a | culture/ PCR (*gltA*)/ IFA | [138] |
|  |  |  | 0% | 3 | n/a | culture/ PCR (*gltA*)/ IFA | [116] |
| ***Microtus socialis*** | Cricetidae | Georgia | 83.3 % | 6 | *B. elizabethae, B. grahamii* | culture/ PCR (*gltA*) | [99] |
|  |  | Israel | 0% | 3 | n/a | PCR (*16S-23S rRNA*/ *gltA*/ *rpoB*) | [49] |
|  |  | Turkey | 57.1 % | 42 | *B. grahamii, B. taylorii* | culture/ PCR (*16S-23S rRNA/ gltA*) | [139] |
| ***Mus booduga*** | Muridae | Sri Lanka | 0% | 4 | n/a | PCR (*nuoG/ gltA/ 16S-23S rRNA*) | [106] |
| ***Mus caroli*** | Muridae | Japan | 0% | 7 | n/a | culture/ PCR (*gltA*/ *rpoB*) | [78] |
|  |  |  | 0% | 1 | n/a | culture/ PCR (*gltA/ rpoB*) | [70] |
|  |  | Laos | 0-50 % | 3 | *B. phoceensis* | culture/ PCR (*16S-23S rRNA*/ *gltA*/ *rpoB*) | [109] |
|  |  |  | 4.8 % | 23 | n/a | culture/ PCR (*16S-23S rRNA*/ *gltA*) | [108] |
|  |  | Taiwan | 100% | 4 | *B. phoceensis, B. tribocorum* | culture/ PCR (*gltA/ rpoB*) | [70] |
|  |  | Thailand | 0% | 3 | n/a | culture/ PCR (*gltA*) | [110] |
|  |  |  | 0% | 5 | n/a | culture/ PCR (*gltA*) | [111] |
|  |  |  | 8% | 26 | n/a | culture/ PCR (*16S-23S rRNA*/ *gltA*) | [108] |
|  |  |  | 0% | 1 | n/a | culture/ PCR *(gltA/* ssrA) | [112] |
| ***Mus cervicolor*** | Muridae | Laos | 0-16.7 % | 71 | Lao/Nh2 | PCR (*16S-23S rRNA*/ *gltA*/ *rpoB*) | [109] |
|  |  | Thailand | 42.9 % | 7 | *B. coopersplainensis, B. tribocorum* | culture/ PCR (*gltA*) | [110] |
|  |  |  | 20.8 % | 48 | unknown *B.* spp. | culture/ PCR (*16S-23S rRNA*/ *gltA*) | [108] |
|  |  |  | 0% | 3 | n/a | culture/ PCR *(gltA/* ssrA) | [112] |
|  |  |  | 0% | 2 | n/a | culture/ PCR *(gltA/* *rpoB*) | [113] |
|  |  |  | 50% | 2 | *B. queenslandensis* | PCR (*16S-23S rRNA*/ *23S rRNA*/ *gltA*/ *ftsZ*/ *rpoB*) | [114] |
| ***Mus cookii*** | Muridae | Laos | 30% | 55 | unknown *B.* spp. | culture / PCR (*16S-23S rRNA*/ *gltA*) | [108] |
|  |  | Thailand | 17.1% | 35 | unknown *B.* spp. | culture / PCR (*16S-23S rRNA*/ *gltA*) | [108] |
| ***Mus domesticus*** | Muridae | Spain | 8.8 % | 34 | *B. taylorii, B. vinsonii* subsp. *vinsonii* | PCR (*16S-23S rRNA*/ *16S rRNA*/ *gltA*) | [90] |
| ***Mus macedonicus*** | Muridae | Bulgaria | 43.2% | 37 | unknown *B.* spp. | blood smear | [59] |
| ***Mus minutoides*** | Muridae | Congo | 66.7 % | 6 | *B. tribocorum* | PCR (*gltA*/ *rpoB*) | [101] |
|  |  | Egypt | 0% | 20 | n/a | culture/ PCR (*gltA*) | [47] |
|  |  | Kenya | 0% | 1 | n/a | culture/ PCR (*gltA*) | [125] |
|  |  | Tanzania | 0% | 3 | n/a | PCR (*ssrA/ gltA*) | [52] |
|  |  |  | 0% | 11 | n/a | PCR (*gltA*/ *rpoB*) | [101] |
| ***Mus musculus*** | Muridae | Argentina | 0% | 85 | n/a | PCR (*16S-23S rRNA*/ *gltA*) | [127] |
|  |  | Bangladesh | 0% | 12 | n/a | culture/ PCR (*gltA*) | [105] |
|  |  | China | 33.3% | 6 | n/a | PCR (*gltA*) | [55] |
|  |  |  | 0% | 39 | n/a | PCR (*gltA*/ *rpoB*) | [61] |
|  |  |  | 0% | 17 | n/a | culture / PCR (*gltA/ ftsZ/ rpoB/ ribC*) | [56] |
|  |  |  | n/a | n/a | *B. elizabethae, B. queenslandensis, B. tribocorum* | PCR (*gltA*) | [140] |
|  |  |  | 0% | 2 | n/a | culture/ PCR (*gltA*) | [57] |
|  |  | Gabon | 0% | 29 | n/a | PCR (*16S-23S rRNA*) | [122] |
|  |  | Israel | 16% | 6 | unknow*n B*. spp. | culture/ PCR (*ssrA/ gltA/ rpoB/ 16S–23S rRN*A) | [48] |
|  |  |  | 0% | 25 | n/a | PCR (*16S-23S rRNA*/ *gltA*/ *rpoB*) | [49] |
|  |  | Kenya | 0% | 178 | n/a | culture/ PCR (*gltA*) | [125] |
|  |  | Lithuania | 8.3 % | 12 | *B. grahamii* | PCR (*16S-23S rRNA*/ *rpoB*/ *groEL*/ *ssrA*) | [67] |
|  |  | Nigeria | 3.4 % | 177 | n/a | PCR (*gltA*) | [120] |
|  |  | Peru | 0% | 3 | n/a | culture/ PCR (*gltA*) | [141] |
|  |  | South Korea | 0% | 1 | n/a | PCR (*23S rRNA*/ *groEL*) | [75] |
|  |  |  | 0% | 2 | n/a | culture/ PCR (*gltA*) | [74] |
|  |  | Sweden | 5.6 % | 18 | *B. grahamii* | culture/ PCR (*gltA*) | [91] |
|  |  | Taiwan | 0% | 2 | n/a | culture/ PCR (*16S-23S rRNA*/ *gltA*)/ IFA | [142] |
|  |  |  | 0% | 2 | n/a | culture/ PCR (*16S-23S rRNA*/ *gltA*/ *ftsZ*/ *rpoB*) | [143] |
|  |  | Tanzania | 0% | 44 | n/a | PCR (*ssrA/ gltA*) | [52] |
|  |  | USA | 0% | 1 | n/a | culture/ PCR (*gltA*) | [119] |
|  |  |  | 0% | 1 | n/a | PCR (*ssrA/ 16S-23S rRNA/ gltA*) | [128] |
|  |  |  | 0% | 9 | n/a | culture/ PCR (*gltA*)/ IFA | [138] |
| ***Mus musculus castaneus*** | Muridae | Nepal | 0% | 3 | n/a | PCR (*gltA*/ *rpoB*) | [107] |
|  |  | Senegal | 4.8% | n/a | n/a | PCR (*16S rRNA*) | [134] |
| ***Mus nannomys*** | Muridae | Gabon | 0% | 22 | n/a | PCR (*16S-23S rRNA*) | [122] |
| ***Mus* spp*.*** | Muridae | Benin | 0% | 1 | n/a | PCR (*rpoB/ gltA*) | [124] |
|  |  | Ethiopia | 5.7 % | 14 | unknown *B.* spp*.* | PCR (*rpoB*) | [100] |
|  |  | Thailand | 75% | 4 | unknown *B.* spp*.* | culture/ PCR (*16S-23S rRNA*/ *gltA*) | [108] |
| ***Mus spretus*** | Muridae | Algeria | 22.2 % | 7 | unknown *B.* spp*.* | PCR (*16S-23S rRNA*/ *ftsZ*) | [104] |
|  |  | Spain | 3.3 % | 30 | *B. elizabethae* | PCR (*16S-23S rRNA*/ *16S rRNA*/ *gltA*) | [90] |
|  |  |  | n/a | 87 | unknown *B.* spp*.* | PCR (*16S-23S rRNA*) | [144] |
| ***Muscardinus avellanarius*** | Gliridae | Croatia | 0% | 1 | n/a | PCR (hbpA/ *gltA/ 16S rRNA*) | [63] |
| ***Myodes (Clethrionomys) gapperi*** | Cricetidae | Canada | 70% | 10 | *B. grahamii, B. elizabethae, B. vinsonii* subsp*. vinsonii* | culture/ PCR (*gltA*) | [145] |
|  |  | USA | 0% | 6 | n/a | culture/ PCR (*gltA*)/ IFA | [138] |
| ***Myodes (Clethrionomys) gapperi / regulus*** | Cricetidae | South Korea | 0% | 1 | n/a | culture/ PCR (*gltA*) | [74] |
| ***Myodes (Clethrionomys) glareolus*** | Cricetidae | Austria | 2.5% | 40 | *B. doshiae* | PCR (*16S-23S rRNA*/ *rpoB)* | [82] |
|  |  | Croatia | 11.6 % | 43 | *B. grahamii* | PCR (hbpA/ *gltA/ 16S rRNA*) | [63] |
|  |  | Denmark | 0% | 15 | n/a | culture/ PCR (*16S rRNA*) | [83] |
|  |  | France | 56.4 % | 447 | *B. doshiae, B. grahamii, B. taylorii, B. rochalimae*-like, unknown *B*. spp. | PCR (*gltA*) | [146] |
|  |  |  | 39.3 % | 471 | *B. doshiae, B. grahamii, B. taylorii, B. rochalimae*-like | PCR (*gltA*/ *ftsZ*/ *groEL*/ *rpoB*/ *ribC*/ *nuoG*/ *virB5*) | [94] |
|  |  | Germany | 52.8 % | 36 | *B. grahamii, B. taylorii,* unknown *B*. spp. | PCR (*16S-23S rRNA*) | [64] |
|  |  |  | 79.8% | 104 | unknown *B.* spp. | PCR (*16S-23S rRNA*) | [65] |
|  |  |  | 47.5% | 395 | *B. grahamii, B. taylorii, B. doshiae,* uknown *B.* spp. | PCR (*16S-23S rRNA/ gltA*) | [66] |
|  |  | Lithuania | 15.5% | n/a | *B. grahamii, B. rochalimae, B. taylorii* | PCR (*16S-23S rRNA*/ *gltA*) | [85] |
|  |  |  | 42.4 % | 165 | *B. grahamii, B. rochalimae, B. taylorii* | PCR (*16S-23S rRNA*/ *rpoB*/ *groEL*/ *ssrA*) | [67] |
|  |  | Poland | 16% | 414 | *B. grahamii, B. taylorii* | PCR (*gltA*) | [86] |
|  |  |  | 31% | 181 | *B. grahamii* | PCR (*gltA*) | [87] |
|  |  |  | 29.1 % | 658 | n/a | PCR (*gltA*) | [88] |
|  |  | Slovakia | 69% | 232 | *B. grahamii, B. taylorii* | PCR (*16S-23S rRNA*) | [89] |
|  |  | Slovenia | 31.7 % | 41 | *B. doshiae, B. birtlesii, B. taylorii* | PCR (*16S-23S rRNA/ ftsZ*) | [73] |
|  |  | Spain | 18.8 % | 16 | *B. taylorii* | PCR (*16S-23S rRNA*/ *16S rRNA*/ *gltA*) | [90] |
|  |  | Sweden | 15% | 60 | *B. grahamii* | culture/ PCR (*gltA*) | [91] |
|  |  | Switzerland | 17.1 % | 129 | *B. grahamii, B. taylorii, Candidatus* B. rudakovii | PCR (*gltA*/ *rpoB/ 16S-23S rRNA*) | [93] |
|  |  | Turkey | 50% | 42 | *B. taylorii, B. grahamii* | culture/ PCR *(gltA*) | [92] |
|  |  | UK | n/a | n/a | *B. doshiae, B. grahamii, B. taylorii* | PCR (*gltA*) | [96] |
|  |  |  | n/a | 1 | *B. grahamii* | PCR (*16S rRNA/ ftsZ/ gltA/ groEL/ ribC/ rpoB*) | [79] |
|  |  |  | 0.6-38.7% | 346 | *B. grahamii, B. birtlesii, B. taylorii, B. doshiae,* unknown *B.* spp. | PCR | [97] |
|  |  |  | 48.9% | 751 | *B. grahamii, B. taylorii, B. birtlesii,B. doshiae, B. rochalimae*-like | PCR (*16S-23S rRNA*) | [98] |
|  |  | Ukraine | 15.4 % | 13 | n/a | PCR (*rpoB*) | [77] |
| ***Myodes (Clethrionomys) rufocanus*** | Cricetidae | Russia | 60% | 15 | *B. taylorii* | PCR (*gltA*) | [69] |
|  |  |  | 17% | 58 | *B. grahamii, B. taylorii,* unkown *B.* spp. | culture/ PCR (*gltA/ rpoB*) | [70] |
| ***Myodes (Clethrionomys) rufocanus* subsp. *bedfordiae*** | Cricetidae | Japan | 23.5 % | 17 | *B. taylorii* | culture/ PCR (*gltA*/ *rpoB*) | [78] |
|  |  |  | 23.5 % | 17 | *B. taylorii* | culture/ PCR (*gltA*/ *rpoB*) | [81] |
|  |  |  | 13% | 8 | *B. taylorii* | culture/ PCR (*gltA/ rpoB*) | [70] |
| ***Myodes (Clethrionomys) rutilus*** | Cricetidae | China | 54.3% | 46 | *B. doshiae, B. grahamii, B. taylorii, B. washoensis* subsp*. cynomysii* | PCR (*gltA/ 16S rRN/ ftsZ/ rpoB*) | [62] |
|  |  | Russia | 50% | 8 | *B. grahamii, B. taylorii* | culture/ PCR (*gltA/ rpoB*) | [70] |
|  |  | USA | 14% | 48 | *B. henselae, B. queenslandensis, B. washoensis, B. vinsonii* subsp. *arupensis* | culture/ PCR (*gltA*/ *rrs*/ *rpoB*) | [147] |
|  |  | Japan | 0% | 1 | n/a | culture/ PCR (*gltA/ rpoB*) | [70] |
| ***Myodes andersoni*** | Cricetidae | Japan | 29% | 7 | *B. taylorii* | culture/ PCR (*gltA/ rpoB*) | [70] |
| ***Neotoma albigula*** | Cricetidae | Mexico | 75% | 4 | unknow*n B*. spp. | PCR (*gltA*) | [118] |
|  |  | USA | 39% | 50 | *B. vinsonii* subsp. vi*nsonii, B. vinsonii* subsp*. arupensis, B. grahamii, B. rochalimae* | PCR (*ssrA/ 16S-23S rRNA/ gltA*) | [128] |
|  |  |  | 73.1 % | 175 | *B. grahamii* | culture/ PCR (*gltA*) | [148] |
| ***Neotoma cineria*** | Cricetidae | USA | 0% | 1 | n/a | culture/ PCR (*16S rRNA*/ *gltA*/ *groEL*) | [129] |
| ***Neotoma micropus*** | Cricetidae | USA | 50% | 3 | unknown *B*. spp. | culture/ PCR (*gltA*) | [119] |
|  |  |  | 74.3% | 272 | *B. vinsonii* subsp .vi*nsonii, B. grahamii, B. rochalimae* | PCR (*ssrA/ 16S-23S rRNA/ gltA*) | [128] |
|  |  |  | 64.2 % | 522 | *B. grahamii* | culture/ PCR (*gltA*) | [148] |
| ***Niviventer confucianus*** | Muridae | China | 39.1% | 64 | *B. grahamii* | PCR (*gltA*) | [55] |
|  |  |  | 4% | 28 | *B. grahamii* | PCR (*gltA*) | [60] |
|  |  |  | 12.5 % | 8 | *B. queenslandensis* | PCR (*gltA*/ *rpoB*) | [61] |
| ***Niviventer cremoriventer*** | Muridae | Malaysia | 35.7% | 14 | n/a | PCR (*gltA*) | [135] |
| ***Niviventer fulvescens*** | Muridae | Cambodia | 10% | 10 | *B. queenslandensis* | culture/ PCR (*16S-23S rRNA*/ *gltA*) | [108] |
|  |  | Laos | 0% | 1 | n/a | culture/ PCR (*16S-23S rRNA*/ *gltA*) | [108] |
|  |  | Thailand | 12.5% | 8 | *B. queenslandensis* | culture/ PCR (*16S-23S rRNA*/ *gltA*) | [108] |
| ***Niviventer* spp.** | Muridae | Malaysia | 0% | 1 | n/a | PCR (*gltA*) | [135] |
| ***Ochotona curzoniae*** | Ochotonidae # | China | 21.7% | 286 | *B. grahamii, B. queenslandensis* | PCR (*gltA/ rpoB*) | [149] |
|  |  |  | 0% | 10 | n/a | culture / PCR (*gltA/ ftsZ/ rpoB/ ribC*) | [56] |
|  |  |  | 19% | 79 | *B. grahamii, B. taylorii* | culture/ PCR (*gltA*) | [150] |
| ***Ochotona daurica*** | Ochotonidae # | China | 20% | 15 | n/a | PCR (*gltA*) | [55] |
| ***Ochrotomys nuttalli*** | Cricetidae | USA | 25% | 4 | unknown *B*. spp. | culture/ PCR (*gltA*)/ IFA | [138] |
| ***Octodon degus*** | Octododidae | Netherlands/ Czech Republic | 0% | 29 | n/a | culture/ PCR (*gltA*) | [47] |
| ***Oligoryzomys favenscens*** | Cricetidae | Argentina | 45.3 % | 86 | *B. grahamii, B. tribocorum,* unknown *B.* spp. | PCR (*16S-23S rRNA*/ *gltA*) | [127] |
| ***Ondatra zibethicus*** | Cricetidae | Belgium | 100% | 1 | *B. grahamii* | PCR (*16S-23S rRNA*) | [36] |
| ***Onychomys arenicola*** | Cricetidae | Mexico | 80.4% | 56 | unknow*n B*. spp. | PCR (*gltA*) | [118] |
| ***Onychomys leucogaster*** | Cricetidae | Mexico | 83.3% | 12 | unknow*n B*. spp. | PCR (*gltA*) | [118] |
|  |  | USA | 73.5 % | 242 | *B. grahamii*-like, *B. vinsonii* subsp. *arupensis*-like, *B. washoensis*-like | culture/ PCR (*gltA*) | [119] |
|  |  |  | 87.5% | 8 | *B. vinsonii* subsp. vi*nsonii* | PCR (*ssrA/ 16S-23S rRNA/ gltA*) | [128] |
| ***Oryctolagus cuniculus*** | Leporidae # | Netherlands | 9.8% | 143 | *B. alsatica,* unknown *B.* spp. | PCR (*gltA*) | [151] |
|  |  | Spain | 17.2% | 279 | *B. alsatica* | PCR (*16S-23S rRNA*/ *gltA*) | [152] |
|  |  |  | 38.5% | 26 | *B. alsatica* | PCR (*16S-23S rRNA*) | [153] |
| ***Oryzomys palustris*** | Cricetidae | USA | 27.6 % | 36 | unknown *B*. spp. | culture/ PCR (*gltA*)/ IFA | [138] |
|  |  |  | 37.5 % | 8 | n/a | culture/ PCR (*gltA*)/ IFA | [116] |
| ***Otomys irroratus*** | Muridae | South Africa | 50% | 2 | n/a | culture/ PCR (*gltA*) | [54] |
| ***Otomys* sp*.*** | Muridae | Congo | 0% | 1 | n/a | PCR (*gltA*/ *rpoB*) | [101] |
| ***Otospermophilus variegatus*** | Sciuridae | USA | 37% | 27 | *B. washoensis* | PCR (*ssrA/ 16S-23S rRNA/ gltA*) | [128] |
| ***Pachyuromys duprasi*** | Muridae | Egypt, Thailand or the Netherlands | n/a | n/a | *B. pachyuromydis* | PCR (*16S rRNA/ ftsZ/ gltA/ rpoB/ 16S–23S rRNA*) | [51] |
|  |  | Netherlands | 72.2 % | 18 | *B. elizabethae*-like, unknown *B.* spp. | culture/ PCR (*gltA*) | [47] |
| ***Paraxerus flavovottis*** | Sciuridae | Tanzania | 33.3 % | 3 | *B. quintana-like* | PCR (*ssrA/ gltA*) | [52] |
| ***Pedetes capensis*** | Pedetidae | South Africa | 0% | 16 | n/a | culture/ PCR (*gltA*) | [54] |
| ***Perognathus flavus*** | Heteromyidae | Mexico | 0% | 6 | unknow*n B*. spp. | PCR (*gltA*) | [118] |
|  |  | USA | 0% | 4 | n/a | culture/ PCR (*gltA*) | [119] |
|  |  |  | 0% | 1 | n/a | PCR (*ssrA/ 16S-23S rRNA/ gltA*) | [128] |
| ***Peromyscus gossypinus*** | Cricetidae | USA | 27.3 % | 11 | unknown *B*. spp. | culture/ PCR (*gltA*)/ IFA | [138] |
| ***Peromyscus leucopus*** | Cricetidae | Mexico | 50% | 16 | unknow*n B*. spp. | PCR (*gltA*) | [118] |
|  |  | USA | 57.1 % | 7 | unknown *B*. spp. | culture/ PCR (*gltA*) | [119] |
|  |  |  | 48% | 25 | *B. vinsonii* subsp. *arupensis* | PCR (*ssrA/ 16S-23S rRNA/ gltA*) | [128] |
|  |  |  | 43.6 % | 96 | unknown *B*. spp. | culture/ PCR (*gltA*)/ IFA | [138] |
|  |  |  | 1.2 % | 81 | n/a | culture/ PCR (*gltA*)/ IFA | [116] |
| ***Peromyscus maniculatus*** | Cricetidae | Canada | 53% | 15 | *B. vinsonii* subsp*. arupensis* | culture/ PCR (*gltA*) | [145] |
|  |  | Mexico | 50% | 24 | unknow*n B*. spp. | PCR (*gltA*) | [118] |
|  |  | USA | 44.4 % | 471 | unknown *B*. spp. | culture/ PCR (*gltA*) | [119] |
|  |  |  | 82.4 % | 737 | *B. vinsonii* subsp. *arupensis* | culture/ PCR (gltA) | [154] |
|  |  |  | 21.4% | 14 | *B. vinsonii* subsp. *arupensis* | PCR (*ssrA/ 16S-23S rRNA/ gltA*) | [128] |
|  |  |  | 48.9 % | 79 | unknown *B*. spp. | culture/ PCR (*gltA*)/ IFA | [138] |
|  |  |  | 53.3 % | 29 | *B. vinsonii*-like, unknown *B*. spp. | culture/ PCR (*16S rRNA*/ *gltA*/ *groEL*) | [129] |
| ***Peromyscus polionotus*** | Cricetidae | USA | 0% | 3 | n/a | culture/ PCR (*gltA*)/ IFA | [138] |
| ***Peromyscus truei*** | Cricetidae | USA | 16.7% | 12 | *B. vinsonii* subsp. *berkhoffii* | PCR (*ssrA/ 16S-23S rRNA/ gltA*) | [128] |
| ***Petinomys phayrei*** | Sciuridae | Laos | 0% | 2 | n/a | PCR (*16S-23S rRNA*/ *gltA*/ *rpoB*) | [115] |
| ***Phodopus roborovskii*** | Cricetidae | China | 14.3% | 7 | n/a | PCR (*gltA*) | [55] |
|  |  |  | 0% | 11 | n/a | culture / PCR (*gltA/ ftsZ/ rpoB/ ribC*) | [56] |
| ***Phyllotis peruviana*** | Cricetidae | Peru | 100% | 2 | unknown *B*. spp. | culture/ PCR (*gltA*) | [141] |
| ***Praomys daltoni*** | Muridae | Mali | 12.5% | 8 | *B. florencae,* unknown *B.* spp. | PCR (*gltA/ rpoB/ ftsZ/ 16S-23S rRNA*) | [123] |
| ***Praomys delectorum*** | Muridae | Tanzania | 66.7% | 9 | *B. elizabethae, B. grahamii* | PCR (*gltA*/ *rpoB*) | [101] |
| ***Praomys sp.*** | Muridae | Gabon | 0% | 17 | n/a | PCR (*16S-23S rRNA*) | [122] |
| ***Psammomys obesus*** | Muridae | Algeria | 28.5 % | 7 | unknown *B.* spp*.* | PCR (*16S-23S rRNA*/ *ftsZ*) | [104] |
|  |  | Egypt | 60% | 10 | *B. elizabethae*-like, unknown *B.* spp. | culture/ PCR (*gltA*) | [47] |
|  |  | Tunisia | 49.1 % | 383 | n/a | n/a | [155] |
| ***Pteromys volans*** | Sciuridae | China | 50% | 10 | *B. grahamii*-like | culture/ PCR (*gltA*) | [47] |
| ***Rattus andamanensis*** | Muridae | Laos | 40% | 5 | *B. rattimassiliensis* | culture/ PCR (*16S-23S rRNA*/ *gltA*) | [108] |
| ***Rattus argentiventer*** | Muridae | Cambodia | 9.5 % | 42 | *B. coopersplainensis, B. queenslandensis, B. rattimassiliensis* | culture/ PCR (*16S-23S rRNA*/ *gltA*) | [108] |
|  |  | Laos | 0% | 1 | n/a | PCR (*16S-23S rRNA*/ *gltA*/ *rpoB*) | [109] |
|  |  | Thailand | 66.7 % | 3 | *B. rattimassiliensis* | culture/ PCR (*gltA*) | [110] |
|  |  |  | 0% | 2 | *B. coopersplainensis, B. queenslandensis, B. rattimassiliensis* | culture/ PCR (*16S-23S rRNA*/ *gltA*) | [108] |
| ***Rattus berdmorei*** | Muridae | Thailand | 0% | 1 | n/a | culture/ PCR *(gltA/* ssrA) | [112] |
|  |  |  | 25% | 20 | *B. coopersplainensis, B. queenslandensis* | PCR (*23S rRNA*/ *gltA*/ *ftsZ*/ *16S-23S rRNA*/ *rpoB*) | [114] |
| ***Rattus bukit*** | Muridae | Thailand | 0% | 3 | n/a | culture/ PCR *(gltA/* ssrA) | [112] |
| ***Rattus bukit bukit*** | Muridae | Thailand | 50% | 2 | *B. rattimassiliensis* | culture/ PCR *(gltA/* *rpoB*) | [113] |
| ***Rattus conatus*** | Muridae | Australia | n/a | 2 | *B. coopersplainensis, B. queenslandensis, B. rattaustraliani* | PCR (*gltA*/ *16S rRNA*/ *ftsZ*/ *16S–23S rRNA*) | [136] |
| ***Rattus diardii*** | Muridae | Malaysia | 13.7 % | 58 | *B. coopersplainensis, B. elizabethae, B. queenslandensis, B. rattimassiliensis* | PCR (*gltA*/ *rpoB*) | [156] |
| ***Rattus exulans*** | Muridae | Cambodia | 3.5 % | 115 | *B. elizabethae, B. queenslandensis, B. tribocorum* | culture/ PCR (*16S-23S rRNA*/ *gltA*) | [108] |
|  |  | Laos | 0-30.4 % | 80 | *B. elizabethae, B. phoceensis, B. tribocorum,* Lao⁄Nh2 | PCR (*16S-23S rRNA*/ *gltA*/ *rpoB*) | [109] |
|  |  |  | 0% | 97 | *B. elizabethae, B. phoceensis, B. tribocorum,* Lao⁄Nh2 | culture/ PCR (*16S-23S rRNA*/ *gltA*/ *rpoB*) | [108] |
|  |  | Myanmar | 21.3 % | 150 | *B. kosoyi,* unknown *B*. spp. | PCR (*nuoG/ gltA/ 16S-23S rRNA*) | [106] |
|  |  | Sri Lanka | 0% | 5 | unknown *B.* spp. | PCR (*nuoG/ gltA/ 16S-23S rRNA*) | [106] |
|  |  | Thailand | 3.2 % | 95 | *B. elizabethae, B. tribocorum* | culture/ PCR (gltA) | [110] |
|  |  |  | 39.5 % | 43 | *B. elizabethae, B. tribocorum* | culture/ PCR (*gltA*/ *16S-23S rRNA*) | [157] |
|  |  |  | 0% | 2 | n/a | culture/ PCR (*gltA*) | [111] |
|  |  |  | 4.2 % | 142 | *B. elizabethae, B. queenslandensis, B. tribocorum* | culture/ PCR (*gltA*/ *16S-23S rRNA*) | [108] |
|  |  |  | 3.1% | 96 | n/a | culture/ PCR *(gltA/* ssrA) | [112] |
|  |  |  | 6.3% | 80 | *B. tribocorum* | culture/ PCR *(gltA/* *rpoB*) | [113] |
|  |  |  | 1.5 % | 69 | unknown *B*. spp. | culture/ PCR *(23S rRNA*/ *gltA*/ *ftsZ*/ *16S-23S rRNA*/ *rpoB*) | [114] |
|  |  |  | 56% | 9 | *B. queenslandensis, B. tribocorum, B. elizabethae* | culture/ PCR (*gltA/ rpoB*) | [70] |
| ***Rattus flavipectus*** | Muridae | China | 18.3% | n/a | *B. elizabethae, B. queenslandensis, B. tribocorum* | PCR (*gltA*) | [140] |
| ***Rattus fluvescens*** | Muridae | Thailand | 0% | 8 | n/a | PCR (*23S rRNA*/ *gltA*/ *ftsZ*/ *16S-23S rRNA*/ *rpoB*) | [114] |
| ***Rattus fuscipes*** | Muridae | Australia | n/a | 1 | *B. coopersplainensis, B. queenslandensis, B. rattaustraliani* | PCR (*gltA*/ *16S rRNA*/ *ftsZ*/ *16S–23S rRNA*) | [136] |
| ***Rattus leucopus*** | Muridae | Australia | n/a | 3 | *B. coopersplainensis, B. queenslandensis, B. rattaustraliani* | PCR (*gltA*/ *16S rRNA*/ *ftsZ*/ *16S–23S rRNA*) | [136] |
| ***Rattus losea*** | Muridae | China | 31% | 45 | *B. grahamii* | PCR (*gltA*) | [60] |
|  |  | Laos | 0% | 8 | n/a | culture/ PCR (*16S-23S rRNA*/ *gltA*) | [108] |
|  |  | Taiwan | 66.7 % | 3 | *B. elizabethae, B. grahamii* | culture/ PCR (*16S-23S rRNA*/ *gltA*)/ IFA | [142] |
|  |  |  | 77% | 64 | *B. phoceensis, B. tribocorum, B. rattimassiliensis, B. coopersplainsensis,* unknown *B.* spp. | culture/ PCR (*gltA/ rpoB*) | [70] |
|  |  | Thailand | 0% | 4 | n/a | culture/ PCR (*gltA*) | [110] |
|  |  |  | 18% | 11 | *B. elizabethae*-like, unknown *B.* spp. | culture/ PCR (*gltA*) | [111] |
|  |  |  | 0% | 39 | n/a | culture/ PCR (*gltA*/ *16S-23S rRNA*) | [108] |
|  |  |  | 0% | 3 | n/a | culture/ PCR *(gltA/* ssrA) | [112] |
|  |  |  | 0% | 1 | n/a | culture/ PCR *(gltA/* *rpoB*) | [113] |
|  |  |  | 0% | 13 | n/a | PCR (*23S rRNA*/ *gltA*/ *ftsZ*/ *16S-23S rRNA*/ *rpoB*) | [114] |
| ***Rattus muelleri*** | Muridae | Thailand | 50% | 2 | *B. rattimassiliensis* | culture/ PCR *(gltA/* *rpoB*) | [113] |
| ***Rattus nitidus*** | Muridae | Thailand | 33.3 % | 3 | *B. rattimassiliensis* | culture/ PCR (*gltA*) | [110] |
|  |  |  | 0% | 13 | n/a | culture/ PCR (*16S-23S rRNA*/ *gltA*) | [108] |
|  |  |  | 100% | 1 | *B. rattimassiliensis* | culture/ PCR *(gltA/* *rpoB*) | [113] |
| ***Rattus norvegicus*** | Muridae | Algeria | 0% | 7 | n/a | PCR (*16S-23S rRNA*/ *ftsZ*) | [104] |
|  |  | Austria | 0% | 43 | n/a | PCR (*16S-23S rRNA*) | [158] |
|  |  | Belgium | 37.4 % | 1097 | *B. tribocorum, B. grahamii* | PCR (*16S-23S rRNA*) | [36] |
|  |  |  | 35% | 60 | *B. tribocorum* | PCR (*16S-23S rRNA*) | [158] |
|  |  | Benin | 20.8% | 24 | *B. tribocorum, B. elizabethae, B. rochalimae* | PCR (*rpoB/ gltA*) | [124] |
|  |  | Brazil | 19.2 % | 26 | *B. queenslandensis, B. tribocorum* | culture/ PCR (*gltA*) | [159] |
|  |  | Cambodia | 8.3% | 24 | *B. tribocorum* | culture/ PCR (*16S-23S rRNA*/ *gltA*) | [108] |
|  |  | China | 3.2% | 31 | *B. grahamii* | PCR (*gltA*) | [55] |
|  |  |  | 16% | 43 | *B. grahamii* | PCR (*gltA*) | [60] |
|  |  |  | 13.79 % | 29 | *B. rattimassiliensis, B. tribocorum* | PCR (*gltA*/ *rpoB*) | [61] |
|  |  |  | 13.5 % | n/a | *B. elizabethae, B. queenslandensis, B. tribocorum* | PCR (*gltA*) | [140] |
|  |  |  | 42.9 % | 17 | *B. elizabethae*-like, unknown *B.* spp*.* | culture/ PCR (*gltA*) | [57] |
|  |  | Czech Republic | 0% | 58 | n/a | PCR (*16S-23S rRNA*) | [158] |
|  |  | Denmark | 0% | 11 | n/a | PCR (*16S-23S rRNA*) | [158] |
|  |  | France | 30.3 % | 66 | *B. phoceensis* sp. nov*., B. rattimassiliensis* sp. nov. | PCR (*16S rRNA*/ *gltA*/ *rpoB/ ftsZ/ ribC/ groEL*) | [160] |
|  |  | Germany | 7.5 % | 307 | *B. grahamii, B. tribocorum* | PCR (*16S-23S rRNA*) | [158] |
|  |  | Hungary | 0% | 18 | n/a | PCR (*16S-23S rRNA*) | [158] |
|  |  | Indonesia | 2.0 % | 49 | unknown *B.* spp. | PCR (*gltA*) | [161] |
|  |  | Japan | 0% | 105 | n/a | culture/ PCR (*gltA*/ *rpoB*) | [78] |
|  |  |  | 0% | 9 | n/a | culture/ PCR (*gltA/ rpoB*) | [70] |
|  |  | Kenya | 50% | 10 | *B. elizabethae, B. tribocorum* | culture/ PCR (*gltA*) | [125] |
|  |  | Malaysia | 13.5 % | 37 | *B. elizabethae, B. tribocorum, B. queenslandensis* | PCR (*gltA*/ *rpoB*) | [156] |
|  |  | Mali | 50% | 6 | *B. mastomydis, B. elizabethae* | PCR (*gltA/ rpoB/ ftsZ/ 16S-23S rRNA*) | [123] |
|  |  | Nigeria | 68.4 % | 177 | *B. elizabethae, B. grahamii, B. tribocorum* | PCR (*gltA*) | [120] |
|  |  | Peru | 66% | 4 | *B. elizabethae* | culture/ PCR (*gltA*) | [141] |
|  |  | Portugal | 100% | 2 | unknown *B*. spp. | PCR (*gltA*) | [162] |
|  |  | Russia | 0% | 1 | n/a | culture/ PCR (*gltA/ rpoB*) | [70] |
|  |  | South Africa | 25% | 124 | *B. elizabethae* | culture/ PCR (*16S-23S rRNA*) | [163] |
|  |  | South Korea | 0% | 6 | n/a | culture/ PCR (*gltA*) | [74] |
|  |  | Spain | n/a | 10 | *B. tribocorum* | PCR (*16S-23S rRNA*) | [144] |
|  |  | Switzerland | 0% | 29 | n/a | PCR (*16S-23S rRNA*) | [158] |
|  |  | Taiwan | 52.7 % | 169 | *B. elizabethae, B. grahamii, B. phoceensis, B. rattimassiliensis, B. tribocorum* | culture/ PCR (*16S-23S rRNA*/ *gltA*/ *ftsZ*/ *rpoB*)/ IFA | [142] |
|  |  |  | 8.6 % | 53 | *B. elizabethae* | culture/ PCR (*16S-23S rRNA*/ *gltA*/ *ftsZ*/ *rpoB*) | [143] |
|  |  |  | 0% | 1 | n/a | culture/ PCR (*gltA/ rpoB*) | [70]T |
|  |  |  | 36% | 61 | *B. elizabethae, B. phoceensis, B. queenslandensis, B. rattimassiliensis, B. tribocorum* | culture/ PCR (*16S-23S rRNA*/ *16S rDNA*/ *gltA*/ *ftsZ*/ *rpoB*) | [164] |
|  |  | Thailand | 86.4 % | 22 | *B. elizabethae, B. rattimassiliensis, B. tribocorum* | culture/ PCR (*gltA*) | [110] |
|  |  |  | 100% | 10 | *B. elizabethae, B. tribocorum* | PCR (*16S-23S rRNA*/ *gltA*) | [157] |
|  |  |  | 12.5% | 40 | *B. tribocorum* | culture/ PCR *(gltA/* ssrA) | [112] |
|  |  |  | 25.6% | 78 | *B. tribocorum, B. elizabethae, B. queenslandensis* | culture/ PCR *(gltA/* *rpoB*) | [113] |
|  |  |  | 0% | 3 | n/a | culture/ PCR (*gltA/ rpoB*) | [70] |
|  |  | Turkey | 0% | 6 | n/a | culture/ PCR *(gltA*) | [92] |
|  |  | USA | 19.4 % | 323 | *B. elizabethae, B. queenslandensis, B. rochalimae, B. tribocorum* | PCR (*gltA*) | [162] |
|  |  |  | 67.5 % | 200 | *B. elizabethae, B. queenslandensis, B. rochalimae, B. tribocorum* | culture/ PCR (*gltA*) | [101] |
| ***Rattus rattus*** | Muridae | Algeria | 12.5 % | 16 | unknown *B.* spp*.* | PCR (*16S-23S rRNA*/ *ftsZ*) | [104] |
|  |  | Australia | 75% | 48 | *B. phoceensis*, unknown *B*. spp. | PCR (*16S-23S rRN*) | [165] |
|  |  | Bangladesh | 32.3% | 99 | *B. elizabethae, B. tribocorum* | culture/ PCR (*gltA*) | [105] |
|  |  | Benin | 0% | 110 | n/a | PCR (*rpoB/ gltA*) | [124] |
|  |  | China | n/a | n/a | *B. elizabethae, B. queenslandensis, B. tribocorum* | PCR (*gltA*) | [140] |
|  |  | Congo | 4% | 25 | *B. grahamii* | PCR (*gltA*/ *rpoB*) | [101] |
|  |  | Ethiopia | 1.5 % | 66 | unknown *B.* spp*.* | PCR (*rpoB*) | [100] |
|  |  | Gabon | 1.8 % | 54 | *B. elizabethae* | PCR (*16S-23S rRNA*) | [122] |
|  |  | Israel | 16% | 62 | *B. elizabethae, B. tribocorum* | culture/ PCR (*16S-23S rRNA*/ *gltA*/ *ribC*/ *rpoB*/ *16S*/ *groEL*) | [166] |
|  |  |  | 24% | 79 | *B. elizabethae*-like *, B. tribocorum*-like | PCR (*16S-23S rRNA*/ *gltA*/ *rpoB*) | [49] |
|  |  | Italy | 6.3 % | 16 | *B. coopersplainensis* | PCR (*16S-23S rRNA*) | [158] |
|  |  | Japan | 6.1 % | 297 | *B. elizabethae, B. phoceensis, B. rattimassiliensis, B. tribocorum* | culture/ PCR (*gltA*/ *rpoB*) | [78] |
|  |  |  | 30% | 27 | *B. tribocorum, B. rattimassiliensis* | culture/ PCR (*gltA/ rpoB*) | [70] |
|  |  | Kenya | 13-60 % | 48 | *B. elizabethae, B. tribocorum, B. queenslandensis* | culture/ PCR (*gltA*) | [125] |
|  |  | Laos | 0-20.1 % | 381 | *B. elizabethae, B. phoceensis, B. tribocorum,* Lao⁄Nh1, Lao⁄Nh2 | PCR (*16S-23S rRNA*/ *gltA*/ *rpoB*) | [109] |
|  |  | Madagascar | 58.9 % | 158 | *B. elizabethae, B. phoceensis. B. rattimassiliensis, B. tribocorum* | PCR (*nuoG*) | [167] |
|  |  | Myanmar | 41.6 % | 72 | *B. kosoyi, B. phoceensis,* unknown *B.* spp. | PCR (*nuoG/ gltA/ 16S-23S rRNA*) | [106] |
|  |  | Nepal | 43.3 % | 90 | *B. coopersplainensis, B. elizabethae, B. phoceensis, B. rattimassiliensis, B. tribocorum,* unknown *B.* spp. | PCR (*gltA*/ *rpoB*) | [107] |
|  |  | New Zealand | 16.8 % | 143 | *B. coopersplainensis, B. henselae* | PCR (*16S-23S rRNA*/ *gltA*/ *rpoB)* | [168] |
|  |  | Nigeria | 26% | 177 | *B. elizabethae* | PCR (*gltA*) | [120] |
|  |  | Portugal | 40% | 5 | unknown *B.* spp. | PCR (*gltA*) | [162] |
|  |  | Senegal | 3.6-11.1% | n/a | n/a | PCR (*16S rRNA*) | [134] |
|  |  | Slovenia | 0% | 17 | n/a | PCR (*16S-23S rRNA*) | [158] |
|  |  | South Africa | 0% | 1 | *B. elizabethae* | culture/ PCR (*16S-23S rRNA*/ *gltA*) | [54] |
|  |  | South Korea | 0% | 2 | n/a | PCR (*23S rRNA*/ *groEL*) | [75] |
|  |  | Spain | 7% | 43 | n/a | PCR (*16S-23S rRNA*) | [158] |
|  |  | Sri Lanka | 18.2 % | 433 | *B. kosoyi, B. phoceensis,* unknown *B.* spp. | PCR (*nuoG/ gltA/ 16S-23S rRNA*) | [106] |
|  |  | Taiwan | 10% | 10 | *B. tribocorum* | culture/ PCR (16S-23S rRNA/ gltA)/ IFA | [142] |
|  |  |  | 1.7 % | 3 | *B. tribocorum* | culture/ PCR (*16S-23S rRNA*/ *gltA*/ *ftsZ*/ *rpoB*) | [169] |
|  |  |  | 20% | 5 | *B. queenslandensis* | culture/ PCR (*16S-23S rRNA*/ *16S rDNA*/ *gltA*/ *ftsZ*/ *rpoB*) | [164] |
|  |  | Tanzania | 17% | 317 | *B. elizabethae, B. rochalimae, B. Tribocorum* | PCR (*ssrA/ gltA*) | [52] |
|  |  | Thailand | 65.2 % | 135 | *B. coopersplainensis, B. phoceensis, B. rattimassiliensis, B. tribocorum,* unknown *B.* spp. | culture/ PCR (*gltA*) | [110] |
|  |  |  | 11.1 % | 9 | *B. tribocorum* | PCR (*gltA*/ *16S-23S rRNA*) | [157] |
|  |  |  | 12.5 % | 24 | *B. elizabethae*-like, unknown *B.* spp. | culture/ PCR (*gltA*) | [111] |
|  |  |  | 32.5% | 163 | *R. rattimassiliensis* | culture/ PCR *(gltA/* ssrA) | [112] |
|  |  |  | 12.9% | 116 | *B. tribocorum, B. rattimassiliensis, B. elizabethae, B. queenslandensis* | culture/ PCR *(gltA/* *rpoB*) | [113] |
|  |  |  | 8.2 % | 49 | *B. coopersplainensis, B. phoceensis* | PCR (*23S rRNA*/ *gltA*/ *ftsZ*/ *16S-23S rRNA*/ *rpoB*) | [114] |
|  |  |  | 68% | 34 | *B. phoceensis, B. queenslandensis, B. rattimassiliensis, B. elizabethae* | culture/ PCR (*gltA/ rpoB*) | [70] |
|  |  | Turkey | 12.5% | 8 | *B. coopersplainsensis* | culture/ PCR *(gltA*) | [92] |
|  |  | Uganda | 0.9% | 228 | *B. elizabethae* | PCR (*16S-23S rRNA*) | [102] |
|  |  | USA | 12% | 87 | *B. elizabethae, B. vinsonii* | PCR (*gltA*) | [162] |
| ***Rattus remotus*** | Muridae | Thailand | 50% | 2 | *B. rattimassiliensis* | culture/ PCR (*gltA*) | [110] |
| ***Rattus sabanus*** | Muridae | Thailand | 16.7% | 6 | n/a | culture/ PCR *(gltA/* ssrA) | [112] |
| ***Rattus* spp.** | Muridae | Malaysia | 47.1 % | 187 | *B. phoceensis, B. rattimassiliensis,* unknown *B.* spp. | PCR (*gltA*) | [135] |
|  |  |  | 3.73% | 134 | *B. phoceensis* | PCR (*16S-23S rRNA/ 16S rRNA*) | [170] |
| ***Rattus surifer*** | Muridae | Thailand | 0% | 2 | n/a | culture/ PCR (*gltA*) | [110] |
|  |  |  | 0% | 10 | n/a | culture/ PCR *(gltA/* ssrA) | [112] |
|  |  |  | 24.2 % | 33 | *B. coopersplainensis, B. phoceensis, B. queenslandensis, Candidatus* *B.* thailandensis | PCR (*23S rRNA*/ *gltA*/ *ftsZ*/ *16S-23S rRNA*/ *rpoB*) | [114] |
| ***Rattus tanezumi*** | Muridae | Cambodia | 24.8 % | 117 | *B. coopersplainensis, B. elizabethae, B. phoceensis, B. queenslandensis, B. rattimassiliensis, B. tribocorum* | culture/ PCR (*16S-23S rRNA*/ *gltA*) | [108] |
|  |  | China | 27% | 7 | *B. grahamii* | PCR (*gltA*) | [60] |
|  |  | Indonesia | 10.3 % | 79 | *B. phoceensis,* unknown *B.* spp. | PCR (*gltA*) | [161] |
|  |  | Laos | 21.8 % | 78 | *B. coopersplainensis, B. elizabethae, B. phoceensis, B. queenslandensis, B. rattimassiliensis, B. tribocorum* | culture/ PCR (*16S-23S rRNA*/ *gltA*) | [108] |
|  |  |  | 0% | 13 | *B. coopersplainensis, B. elizabethae, B. phoceensis, B. queenslandensis, B. rattimassiliensis, B. tribocorum* | culture/ PCR (*16S-23S rRNA*/ *gltA*) | [108] |
|  |  | Thailand | 31.6 % | 38 | *B. coopersplainensis, B. elizabethae, B. phoceensis, B. queenslandensis, B. rattimassiliensis, B. tribocorum* | culture/ PCR (*16S-23S rRNA*/ *gltA*) | [108] |
|  |  |  | 28.2% | 39 | *B. rattimassiliensis* | culture/ PCR *(gltA/* *rpoB*) | [113] |
|  |  |  | 20.7 % | 29 | *B. coopersplainensis, B. elizabethae, B. phoceensis, B. queenslandensis, B. rattimassiliensis, B. tribocorum* | culture/ PCR (*16S-23S rRNA*/ *gltA*) | [108] |
| ***Rattus tanezumi flavipectus*** | Muridae | China | 41.4 % | 140 | *B. elizabethae*-like, unknown *B. spp.* | culture/ PCR (*gltA*) | [57] |
| ***Rattus tunneyi*** | Muridae | Australia | n/a | 2 | *B. coopersplainensis, B. queenslandensis, B. rattaustraliani* | PCR (*gltA*/ *16S rRNA*/ *ftsZ*/ *16S–23S rRNA*) | [136] |
| ***Reithrodontomys humulis*** | Cricetidae | USA | 33.3 % | 2 | unknown *B*. spp. | culture/ PCR (*gltA*)/ IFA | [138] |
|  |  |  | 8.3 % | 12 | n/a | culture/ PCR (*gltA*)/ IFA | [116] |
| ***Reithrodontomys megalotis*** | Cricetidae | USA | 0% | 1 | n/a | culture/ PCR (*gltA*) | [119] |
|  |  |  | 50% | 2 | unknown *B.* spp. | PCR (*ssrA/ 16S-23S rRNA/ gltA*) | [128] |
| ***Reithrodontomys montanus*** | Cricetidae | USA | 0% | 6 | n/a | culture/ PCR (*gltA*) | [119] |
| ***Rhabdomys pumilio*** | Muridae | South Africa | 44.4 % | 9 | unknown *B.* spp. | culture/ PCR (*gltA*) | [54] |
| ***Rhizomys pruinosus*** | Spalacidae | Cambodia | 0% | 1 | n/a | culture/ PCR (*16S-23S rRNA*/ *gltA*) | [108] |
| ***Rhizomys sumatrensis*** | Spalacidae | Laos | 0% | 3 | n/a | PCR (*16S-23S rRNA*/ *gltA*/ *rpoB*) | [109] |
| ***Saccostomus campestris*** | Nesomyidae | South Africa | 50% | 2 | *B. elizabethae* | culture/ PCR (*gltA*) | [54] |
| ***Salpingotulus michaelis*** | Dipodidae | Pakistan | 0% | 20 | n/a | culture/ PCR (*gltA*) | [47] |
| ***Scapteromys aquaticus*** | Cricetidae | Argentina | 100% | 4 | *B. grahamii, B. tribocorum,* unknown *B*. spp. | PCR (*16S-23S rRNA*/ *gltA*) | [127] |
| ***Sciurus carolinensis*** | Sciuridae | UK | 20% | 20 | *B. vinsonii*-like | culture/ PCR (*gltA*/ *groEL*) | [171] |
|  |  | USA | 28% | 18 | *B. vinsonii*-like | culture/ PCR (*gltA*/ *groEL*) | [171] |
| ***Sciurus vulgaris*** | Sciuridae | UK | 60% | 20 | unknown *B*. spp. | culture/ PCR (*gltA*/ *groEL*) | [171] |
| ***Sciurus vulgaris* subsp. *orientis*** | Sciuridae | China | 20% | 10 | *B. grahamii*-like, *B. washoensis*-like | culture/ PCR (*gltA*) | [47] |
| ***Sekeetamys calurus*** | Muridae | Egypt | 100% | 10 | *B. elizabethae*-like, unknown *B.* spp. | culture/ PCR (*gltA*) | [47] |
| ***Sigmodon hispidus*** | Cricetidae | USA | 55.9 % | 145 | *B. vinsonii, B. vinsonii* subsp*. berkhoffii,* unknown *B*. spp. | culture/ PCR (*gltA*)/ IFA | [138] |
|  |  |  | 78.5 % | 540 | n/a | culture/ PCR (*gltA*)/ IFA | [116] |
| ***Sorex araneus*** | Soricidae § | Croatia | 16.6 % | 6 | unknown *B.* spp. | PCR (hbpA/ *gltA/ 16S rRNA*) | [63] |
|  |  | Finland | 12.3 % | 65 | n/a | blood smear | [172] |
|  |  | Germany | 0% | 1 | n/a | PCR (*16S-23S rRNA*) | [64] |
|  |  | Sweden | 15% | 20 | *B. taylorii* | culture/ PCR (*gltA*) | [91] |
| ***Sorex caecutiens*** | Soricidae § | Finland | 0% | 21 | n/a | blood smear | [172] |
| ***Sorex coronatus*** | Soricidae § | Spain | 35.7 % | 14 | *B. taylorii, B. vinsonii* subsp. *vinsonii, B. vinsonii* subsp. *arupensis* | PCR (*16S-23S rRNA*/ *16S rRNA*/ *gltA*) | [90] |
| ***Sorex minutus*** | Soricidae § | Finland | 0% | 25 | n/a | blood smear | [172] |
| ***Sorex* spp.** | Soricidae § | Germany | 40% | 5 | n/a | PCR (*16S-23S rRNA/ gltA*) | [66] |
| ***Sorex vulgaris*** | Soricidae § | Denmark | 20% | 5 | *B. birtlesii* | culture/ PCR (*16S rRNA*) | [83] |
| ***Spermophilus alashanicus*** | Sciuridae | China | 7.4% | 27 | *B. washoensis* | PCR (*gltA*) | [55] |
| ***Spermophilus beecheyi*** | Sciuridae | USA | 17.1 % | 41 | *B. washoensis, B. washoensis*-like | culture/ PCR (*16S rRNA*/ *gltA*/ *groEL*) | [129] |
| ***Spermophilus columbianus*** | Sciuridae | North America | 30% | 20 | *B. washoensis*-like | culture/ PCR (*gltA*) | [47] |
| ***Spermophilus dauricus*** | Sciuridae | China | 40% | 10 | *B. washoensis*-like | culture/ PCR (*gltA*) | [47] |
| ***Spermophilus franklinii*** | Sciuridae | Canada | 90% | 10 | *B. washoensis,* unknown *B*. spp. | culture/ PCR (*gltA*) | [145] |
| ***Spermophilus lateralis*** | Sciuridae | USA | 25% | 12 | *B. washoensis*-like | culture/ PCR (*16S rRNA*/ *gltA*/ *groEL*) | [129] |
| ***Spermophilus richardsonii*** | Sciuridae | Canada | 49% | 63 | *B. washoensis,* unknown *B*. spp. | culture/ PCR (*gltA*) | [145] |
|  |  |  | 48% | 368 | n/a | culture/ PCR (*gltA*) | [173] |
|  |  | North America | 60% | 20 | *B. washoensis*-like | culture/ PCR (*gltA*) | [47] |
| ***Spermophilus spilosoma*** | Sciuridae | Mexico | 33.3% | 6 | n/a | PCR (*gltA*) | [118] |
| ***Spermophilus tridecemlineatus*** | Sciuridae | Canada | 75% | 4 | *B. washoensis,* unknown *B*. spp. | culture/ PCR (*gltA*) | [145] |
|  |  | USA | 43.1 % | 67 | unknown *B*. spp. | culture/ PCR (*gltA*) | [119] |
| ***Stenocephalemys albipes*** | Muridae | Ethiopia | 64.4 % | 113 | *B. elizabethae,* unknown *B.* spp*.* | PCR (*rpoB*) | [100] |
| ***Suncus murinus*** | Soricidae § | Bangladesh | 42.9 % | 14 | *B. elizabethae* | culture/ PCR (*gltA*) | [105] |
|  |  | Cambodia | 5.1 % | 39 | *B. queenslandensis* | culture/ PCR (*16S-23S rRNA*/ *gltA*) | [108] |
|  |  | China | 30% | 23 | *B. grahamii* | PCR (*gltA*) | [60] |
|  |  |  | 21.4 % | n/a | *B. elizabethae, B. queenslandensis, B. tribocorum* | PCR (*gltA*) | [140] |
|  |  | Indonesia | 4.8 % | 85 | *B. rattimassiliensis* | PCR (*gltA*) | [161] |
|  |  | Japan | 67% | 24 | unknown *B.* spp. | culture/ PCR (*gltA/ rpoB*) | [70] |
|  |  | Myanmar | 3.6 % | 110 | *B. henselae,* unknown *B*. spp. | PCR (*nuoG/ gltA/ 16S-23S rRNA*) | [106] |
|  |  | Nepal | 64.1 % | 92 | *B. elizabethae, B. queenslandensis, B. rochalimae,* unknown *B.* spp. | PCR (*gltA*/ *rpoB*) | [107] |
|  |  | Sri Lanka | 0% | 43 | n/a | PCR (*nuoG/ gltA/ 16S-23S rRNA*) | [106] |
|  |  | Taiwan | 28.6 % | 126 | *B. rattimassiliensis, B. tribocorum* | culture/ PCR (*16S-23S rRNA*/ *gltA*)/ IFA | [142] |
|  |  |  | 43% | 7 | *B. coopersplainsensis, unknown B. spp.* | culture/ PCR (*gltA/ rpoB*) | [70] |
|  |  |  | 20% | 20 | *B. tribocorum* | culture/ PCR (*16S-23S rRNA*/ *16S rDNA*/ *ftsZ*/ *rpoB*) | [164] |
|  |  | Thailand | 3.8% | 26 | *B. elizabethae* | culture/ PCR *(gltA/* *rpoB*) | [113] |
| ***Suncus varilla*** | Soricidae § | South Africa | 0% | 2 | n/a | culture/ PCR (*gltA*) | [54] |
| ***Sundamys muelleri*** | Muridae | Malaysia | 87% | 100 | *B. phoceensis, B. rattimassiliensis,* unknown *B.* spp. | PCR (*gltA*) | [135] |
| ***Sylvilagus audubonii*** | Leporidae # | USA | 70% | 10 | *B. vinsonii* subsp. *vinsonii, B. alsatica* | PCR (*ssrA/ 16S-23S rRNA/ gltA*) | [128] |
| ***Sylvilagus bachmani riparius*** | Leporidae # | USA | 0% | 41 | n/a | culture | [174] |
| ***Talpa europaea*** | Talpidae § | Germany | 100% | 1 | unknown *B*. spp. | PCR (*16S-23S rRNA*) | [64] |
|  |  |  | 100% | 1 | n/a | PCR (*16S-23S rRNA/ gltA*) | [66] |
|  |  | Spain | 71.4 % | 21 | *B. vinsonii* subsp*. vinsonii,* unknown *B.* spp. | PCR (*16S-23S rRNA*/ *16S rRNA*/ *gltA*) | [90] |
| ***Tamias minimus*** | Sciuridae | Canada | 50% | 2 | *B. washoensis,* unknown *B*. spp. | culture/ PCR (*gltA*) | [145] |
|  |  | USA | 44.0 % | 25 | *B. washoensis*-like | culture/ PCR (*16S rRNA*/ *gltA*/ *groEL*) | [129] |
| ***Tamias sibiricus*** | Sciuridae | China | 41.4 % | 29 | *B. grahamii*-like, *B. washoensis*-like | culture/ PCR (*gltA*) | [47] |
|  |  | Russia | 33% | 3 | unknown *B.* spp. | culture/ PCR (*gltA/ rpoB*) | [70] |
| ***Tamiasciurus hudsonicus*** | Sciuridae | USA | 16.7 % | 18 | *B. washoensis*-like | culture/ PCR (*gltA*) | [47] |
| ***Tatera leucogaster*** | Muridae | South Africa | 63.2 % | 19 | *B. elizabethae, B. grahamii* | culture/ PCR (*gltA*) | [54] |
| ***Thomomys talpoides*** | Geomyidae | USA | 0% | 1 | n/a | culture/ PCR (*gltA*) | [119] |
| ***Tscherskia triton*** | Cricetidae | China | 8.6 % | 35 | *B. grahamii* | PCR (*gltA*/ *rpoB*) | [61] |
|  |  | South Korea | 0% | 2 | n/a | culture/ PCR (*gltA*) | [74] |
| ***Tupaia belangeri*** | Tupaiidae | China | 0% | 4 | n/a | culture/ PCR (*gltA*) | [57] |
| ***Tupaia glis*** | Tupaiidae | Thailand | 0% | 2 | n/a | culture/ PCR (*gltA*) | [111] |
| ***Uromys caudimaculatus*** | Muridae | Australia | n/a | 1 | *B. coopersplainensis, B. queenslandensis, B. rattaustraliani* | PCR (*gltA*/ *16S rRNA*/ *ftsZ*/ *16S–23S rRNA*) | [136] |
| ***Urotrichus talpoides*** | Talpidae | Japan | 0% | 1 | n/a | culture/ PCR (*gltA/ rpoB*) | [70] |
| ***Xerospermophilus spilosoma*** | Sciuridae | USA | 0% | 6 | n/a | PCR (*ssrA/ 16S-23S rRNA/ gltA*) | [128] |

§ order Eulipotyphla.

# order Lagomorpha.

n/a: not available, not determined.
